# The fungal pathogen *Magnaporthe oryzae* suppresses innate immunity by modulating a host potassium channel

**DOI:** 10.1371/journal.ppat.1006878

**Published:** 2018-01-31

**Authors:** Xuetao Shi, Yu Long, Feng He, Chongyang Zhang, Ruyi Wang, Ting Zhang, Wei Wu, Zeyun Hao, Yi Wang, Guo-Liang Wang, Yuese Ning

**Affiliations:** 1 State Key Laboratory for Biology of Plant Diseases and Insect Pests, Institute of Plant Protection, Chinese Academy of Agricultural Sciences, Beijing, China; 2 State Key Laboratory of Plant Physiology and Biochemistry, College of Biological Sciences, China Agricultural University, Beijing, China; 3 Department of Plant Pathology, Ohio State University, Columbus, Ohio, United States of America; Nanjing Agricultural University, CHINA

## Abstract

Potassium (K^+^) is required by plants for growth and development, and also contributes to immunity against pathogens. However, it has not been established whether pathogens modulate host K^+^ signaling pathways to enhance virulence and subvert host immunity. Here, we show that the effector protein AvrPiz-t from the rice blast pathogen *Magnaporthe oryzae* targets a K^+^ channel to subvert plant immunity. AvrPiz-t interacts with the rice plasma-membrane-localized K^+^ channel protein OsAKT1 and specifically suppresses the OsAKT1-mediated K^+^ currents. Genetic and phenotypic analyses show that loss of *OsAKT1* leads to decreased K^+^ content and reduced resistance against *M*. *oryzae*. Strikingly, AvrPiz-t interferes with the association of OsAKT1 with its upstream regulator, the cytoplasmic kinase OsCIPK23, which also plays a positive role in K^+^ absorption and resistance to *M*. *oryzae*. Furthermore, we show a direct correlation between blast disease resistance and external K^+^ status in rice plants. Together, our data present a novel mechanism by which a pathogen suppresses plant host immunity by modulating a host K^+^ channel.

## Author summary

Plant nutritional status can greatly influence plant immunity in response to pathogen invasion. Rice blast, a devastating rice disease caused by the hemibiotrophic fungus *Magnaporthe oryzae*, causes a significant reduction in yield and affects food security. In this study, we demonstrate that the *M*. *oryzae* secreted protein AvrPiz-t interacts with rice OsAKT1, a potassium (K^+^) channel protein, and suppresses OsAKT1-mediated inward K^+^ currents, possibly by competing with the OsAKT1 upstream regulator, OsCIPK23. We also show that both OsAKT1 and OsCIPK23 are required for K^+^ uptake and resistance against *M*. *oryzae* infection in rice. This study provides new insights into the molecular basis of pathogen-mediated perturbation of a plant nutrition pathway.

## Introduction

Potassium (K^+^) plays important roles in many fundamental processes in plants, including enzyme activation, cellular homeostasis, membrane transport, osmoregulation and immunoreaction [1, 2]. The uptake and translocation of K^+^ in plants relies on a number of K^+^ channels and transporters. In the model plant *Arabidopsis* (*Arabidopsis thaliana*), the K^+^ channel AKT1 and the K^+^ transporter HAK5 have been reported to mediate most of the K^+^ absorption [3]. Similar to the regulation of AKT1 in *Arabidopsis*, the activity of its homolog in rice (*Oryza sativa*), OsAKT1, is regulated by a protein complex comprising OsCBL1 (calcineurin B-like protein 1) and the cytosolic protein kinase OsCIPK23 (CBL-interacting protein kinase 23) [4], while OsHAK5 is mainly regulated at the transcription level [5].

In agricultural production, the application of potassium fertilizer has been reported to decrease the incidence of plant diseases [2, 6]. For example, the foliar application of potassium chloride (KCl) reduces damage caused by *Septoria tritici* on wheat (*Triticum aestivum*) in field experiments [7], and the application of KCl to K-deficient soils increases rice resistance to stem rot and aggregate sheath spot [8]. Although previous studies have investigated the effect of K^+^ nutrition on disease development in plants [2], it is not known how host K^+^ nutrition reduces pathogen virulence and enhances host immunity.

Adapted plant pathogens secrete effectors into the apoplast of their host or deliver them inside the host cells to promote infection [9, 10]. These molecules can alter plant processes and target a wide range of host proteins, including important components in pathogen-associated molecular pattern (PAMP)-triggered immunity (PTI), effector-triggered immunity (ETI), vesicle trafficking, autophagy, chloroplast and mitochondrial functions, sugar transport, phytoalexin production, etc. [11–16]. The mechanisms underlying the effector-mediated suppression of host immunity have been extensively studied over the last two decades; however, to date no effector has been reported to manipulate K^+^ transport pathways in plants.

The hemibiotrophic fungus, *Magnaporthe oryzae*, causes rice blast disease in all rice-growing countries [17–19] and also causes wheat blast in South America and Bangladesh [20–22]. We previously identified an *M*. *oryzae* effector protein, AvrPiz-t, which functions as a virulence factor and increases blast susceptibility of rice in the absence of the blast resistance (R) protein Piz-t [23, 24]. Yeast two-hybrid (Y2H) screening of a rice cDNA library revealed that AvrPiz-t interacted with 12 APIPs (AvrPiz-t interacting proteins) in rice [24]. Of these, APIP6 and APIP10, two RING-type E3 ligase proteins, have been shown to ubiquitinate and degrade AvrPiz-t, accompanied by the degradation of these two E3 ligases [24, 25]. APIP6 and APIP10 are positive regulators of PTI, while APIP10 acts as a negative regulator of Piz-t accumulation, thereby modulating ETI [24, 25]. Thus, the targeting of different rice E3 ligases by AvrPiz-t to suppress rice immunity involves a multilayered strategy [26]. Recently, we reported that the bZIP-type transcription factor, APIP5, can also be bound by AvrPiz-t [27]. AvrPiz-t suppresses the transcription and protein accumulation of APIP5 at the necrotrophic stage; however, APIP5 also interacts with the R protein Piz-t, thereby stabilizing APIP5 and preventing effector-triggered necrosis [27].

Here, we report that AvrPiz-t interacts with the rice K^+^ channel protein OsAKT1 and suppresses OsAKT1-mediated inward K^+^ current. AvrPiz-t competes with a cytoplasmic kinase, OsCIPK23, for binding to OsAKT1. Both OsAKT1 and OsCIPK23 serve dual functions on K^+^ absorption and blast disease resistance. In addition, we demonstrate the positive effect of K^+^ on rice blast resistance. Based on these results, we propose a working model wherein the *M*. *oryzae* effector AvrPiz-t suppresses rice immunity by interfering with K^+^ signaling components that are important for both K^+^ absorption and host resistance.

## Results

### The *M*. *Oryzae* effector AvrPiz-t interacts with the rice potassium channel protein OsAKT1

The rice OsAKT1 (APIP7) was one of the 12 APIPs identified in our previous study [24]. OsAKT1 has been identified as an inward-rectifying K^+^ channel and play important roles in K^+^ uptake [4, 28]. OsAKT1 is a typical Shaker family K^+^ channel protein with 6 transmembrane domains (TMs), a putative cyclic nucleotide binding domain (cNMP) and 5 ankyrin repeats (ANKs) in the intracellular domain (S1A Fig). The P-loop between the fifth and sixth TM domains contains a TxxTxGYG motif which is the hall-mark of K^+^-selective channels [29]. OsAKT1 also shares high similarities with its orthologs from other plant species [4].

**Fig 1:**
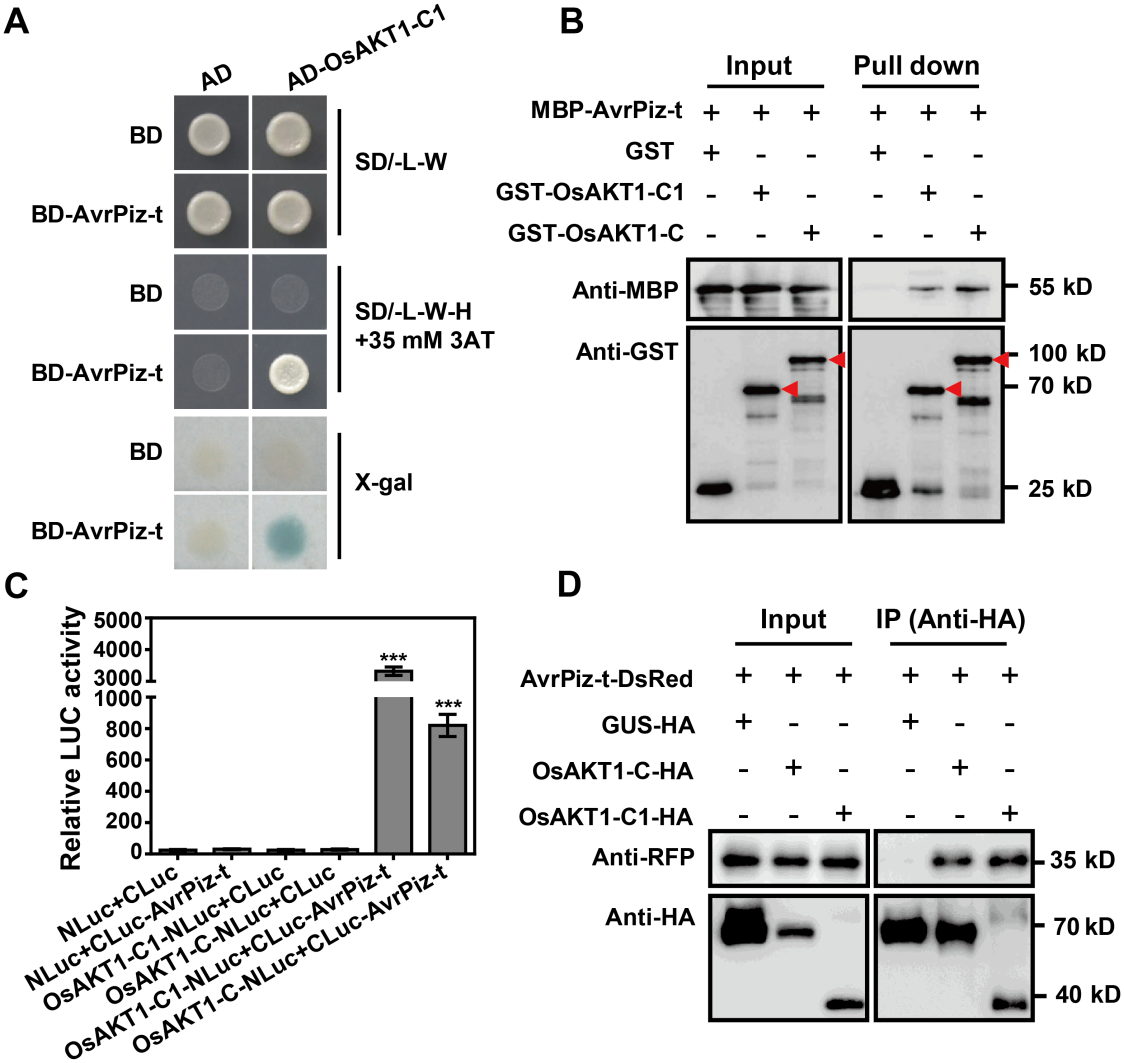
The effector AvrPiz-t from the rice blast pathogen *M*. *oryzae* targets the rice potassium channel protein OsAKT1. (**A**) Y2H analysis of the interaction between BD-AvrPiz-t (without the signal peptide) and AD-OsAKT1-C1. Cells were plated on SD/-Leu-Trp (SD/-L-W) medium. SD/-Leu-Trp-His (SD/-L-W-H) medium containing 35 mM 3-amino-1,2,4-triazole (3AT) and X-gal solution were used to test the interaction. (**B**) GST pull-down assay to confirm the interaction between MBP-AvrPiz-t and GST-OsAKT1-C/C1. GST was used as a negative control. Protein extracts were examined by immunoblot analysis with anti-MBP or anti-GST antibody. Red arrows indicate the expected proteins. (**C**) Luciferase complementation assay was performed to test the interaction between AvrPiz-t and OsAKT1-C/C1 in *N*. *benthamiana* leaves. Leaf disks of *N*. *benthamiana* were used to measure the luminescence 36 h after the infiltration of *Agrobacterium* carrying indicated constructs. Data are shown as means ± s.e.m. (*n* = 3). Student’s *t*-test (***P < 0.001). (**D**) Co-IP assay in *N*. *benthamiana*. *AvrPiz-t-DsRed* and *OsAKT1-C/C1-HA* plasmids were co-expressed in *N*. *benthamiana* leaves following agroinfiltration. The expressed proteins were immunoprecipitated with an anti-HA antibody. Proteins were detected by immunoblot analysis with anti-RFP and Anti-HA antibodies, respectively.

In this study, we first confirmed the interaction between AvrPiz-t and OsAKT1 in yeast. In a Y2H assay, AvrPiz-t (without the signal peptide) interacted with a truncated OsAKT1-C1 fragment (corresponding to amino acids (aa) 607–935 of the full-length protein) derived from a rice cDNA library (Fig 1A). Although we did not detect an interaction between OsAKT1-C (aa 341–935, containing the full intracellular domain) and AvrPiz-t in yeast (S1A and S1B Fig), *in vitro* GST-pull down and *in vivo* luciferase complementation assays demonstrated that both OsAKT1-C1 and OsAKT1-C can bind to AvrPiz-t (Fig 1B and 1C). We then performed a co-immunoprecipitation (Co-IP) assay by transiently expressing *OsAKT1-C-HA* or *OsAKT1-C1-HA* with *AvrPiz-t-DsRed* in *Nicotiana benthamiana* expression system, and confirmed that OsAKT1-C and -C1 fragments interact with AvrPiz-t *in planta* (Fig 1D).

To identify the region of OsAKT1 responsible for the interaction, we performed a Y2H analysis with AvrPiz-t and various truncated versions of an OsAKT1 C-terminal protein fragment. This revealed that the third ANK domain was required for the interaction (S1A and S1B Fig). In addition, we tested the interaction between OsAKT1-C1 and a set of AvrPiz-t point mutants and found that the C70A mutant protein showed a weakened interaction with OsAKT1-C1 (S1C Fig). This point mutation is also important for the binding of AvrPiz-t to APIP5 [27], indicating the important role of this residue in AvrPiz-t’s interactions with rice targets. Collectively, these results demonstrate that AvrPiz-t directly interacts with the OsAKT1 intracellular domain both *in vitro* and *in vivo*.

### AvrPiz-t suppresses OsAKT1-mediated inward K^+^ currents

OsAKT1 can mediate inward K^+^ currents in HEK293 cells [4, 28]. To determine whether AvrPiz-t can affect OsAKT1-mediated inward K^+^ currents, we co-expressed OsAKT1 as a fusion protein with green fluorescent protein (OsAKT1-GFP), together with AvrPiz-t in HEK293 cells. The full-length *OsAKT1* CDS was fused to the N terminus of *GFP* under the control of the human cytomegalovirus (CMV) immediate early promoter (S2A Fig). Because AvrPiz-t is a small effector protein (91aa, without the signal peptide) [23], we expressed it under the CMV promoter without adding a tag that might affect the biochemical function of AvrPiz-t. To monitor the transfection efficiency, we cloned the fragment of the red fluorescent protein (*DsRed*) in the same vector under the control of the eukaryotic translation elongation factor 1α (EF-1α) promoter (S2A Fig). The red fluorescent signals were used as a transfection marker in the electrophysiology assays. The protein expression of OsAKT1-GFP and DsRed in the HEK293 cells was determined with immunoblot analysis (S2B Fig).

**Fig 2:**
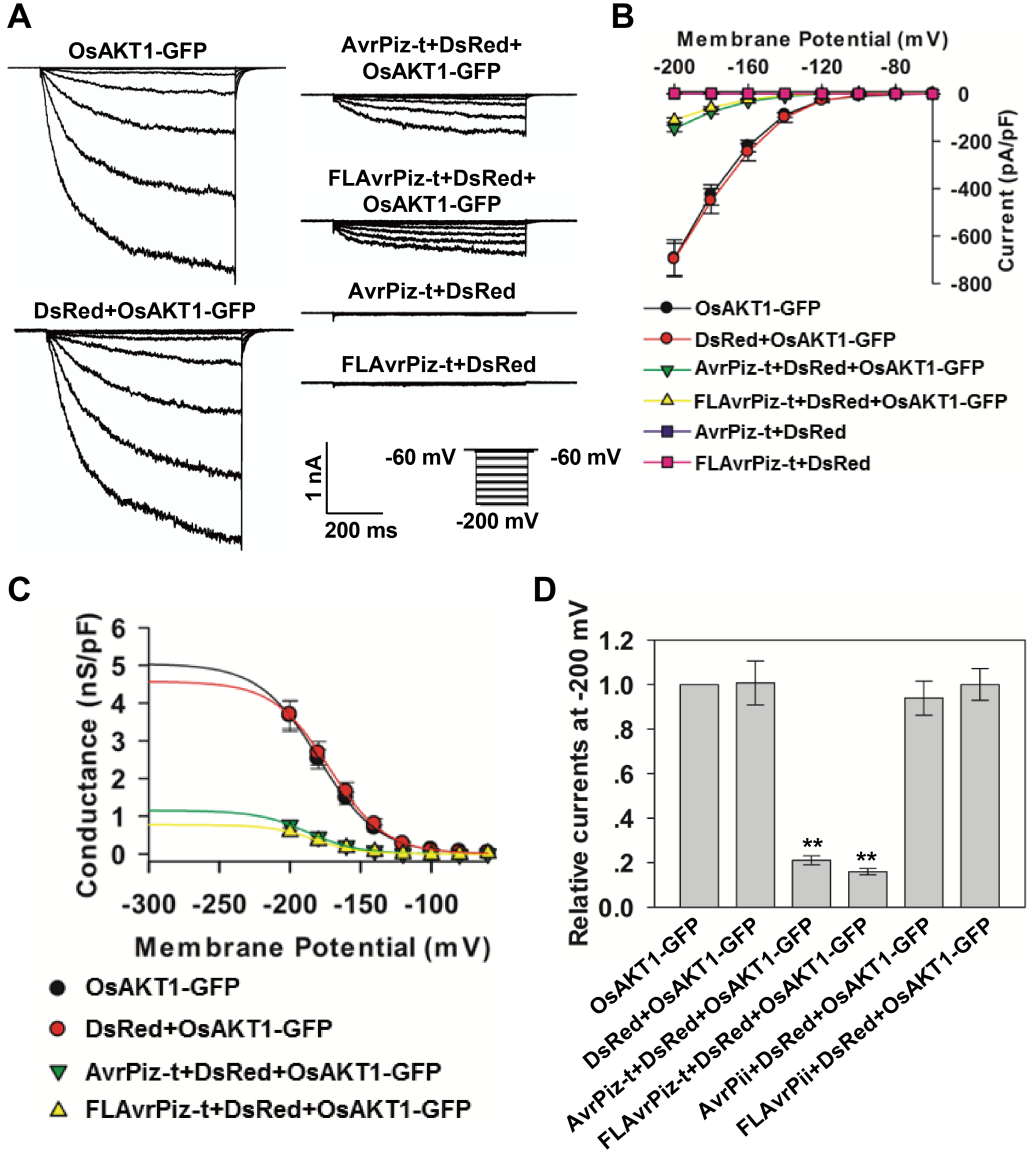
AvrPiz-t suppresses the OsAKT1-mediated inward K^+^ currents. (**A**) Patch-clamp whole-cell recordings of inward K^+^ currents in HEK293 cells expressing indicated constructs. The voltage protocols, as well as time and current scale bars for the recordings are shown. (**B**) The I-V relationship of the steady state whole-cell inward K^+^ currents in HEK293 cells. The data are derived from the recordings as shown in (**A**) and presented as means ± s.e.m. (OsAKT1-GFP, *n* = 25; DsRed+OsAKT1-GFP, *n* = 19; AvrPiz-t+DsRed+OsAKT1-GFP, *n* = 41; FLAvrPiz-t+DsRed+OsAKT1-GFP, *n* = 47; AvrPiz-t+DsRed, *n* = 20; FLAvrPiz-t+DsRed, *n* = 20). (**C**) The G-V relationship of the steady state whole-cell inward K^+^ currents in HEK293 cells. The solid lines represented the best fits of conductance according to the Boltzmann function. The data are derived from the recordings as shown in (**A**) and presented as means ± s.e.m. (**D**) Relative steady state whole-cell inward K^+^ currents in HEK293 cells. The data are derived from the recordings as shown in (**A**) and S3B Fig. Student’s *t*-test (**P < 0.01) was used to analyze statistical significance.

The above assays showed that OsAKT1-GFP alone, or OsAKT1-GFP expressed with the DsRed mediated strong inward K^+^ currents (Fig 2A, left panel); however, the K^+^ currents were substantially suppressed by co-expression with AvrPiz-t or the full-length AvrPiz-t (FLAvrPiz-t) (Fig 2A, top two images of the right panel). In contrast, the combination of AvrPiz-t and DsRed, or FLAvrPiz-t and DsRed, did not mediate any K^+^ currents (Fig 2A, third and fourth images of the right panel). After co-transfection with AvrPiz-t or FLAvrPiz-t, the currents mediated by OsAKT1 decreased from -698±68 pA/pF to -146±14 pA/pF and -111±9 pA/pF, respectively, at -200 mV (Fig 2B). However, the AvrPiz-t and FLAvrPiz-t proteins only inhibited the conductance of OsAKT1, but not the voltage dependence of OsAKT1 (Fig 2C).

To confirm that AvrPiz-t specifically suppresses the OsAKT1-mediated inward K^+^ currents, we used AvrPii, another *M*. *oryzae* effector protein [30], which did not interact with OsAKT1-C1 or OsAKT1-C in Y2H analysis (S3A Fig), in the electrophysiology experiment. We co-expressed AvrPii (without the signal peptide) and the full-length AvrPii (FLAvrPii) with OsAKT1 in HEK293 cells (S2A and S2B Fig), and found neither AvrPii nor FLAvrPii significantly affected the OsAKT1-mediated inward K^+^ currents (Fig 2D, S3B and S3C Fig). These data suggest that AvrPiz-t specifically suppresses OsAKT1-mediated inward K^+^ currents and that this inhibition is dependent on the direct interaction between the two proteins.

### K^+^ uptake is partially blocked in *AvrPiz-t* transgenic plants

A previous study showed that ectopic expression of *AvrPiz-t* in transgenic rice impairs blast resistance and PAMP induced production of reactive oxygen species (ROS) [24]. To investigate whether AvrPiz-t affects the K^+^ absorption in rice, we performed a K^+^-depletion assay and found that *AvrPiz-t* transgenic plants exhibited weaker K^+^ uptake than segregated wild-type (sWT) plants (S4A Fig). K^+^ content analysis showed that *AvrPiz-t* transgenic plants displayed reduced K^+^ level in 0.1 mM K^+^ solution compared to the sWT, while this difference was disappeared when the external K^+^ concentration was elevated to 1.0 mM, suggesting that AvrPiz-t inhibition effect could be restored by the increase of K^+^ supply (S4B Fig). We then measured the plant net K^+^ fluxes using non-invasive micro-test technology (NMT) in the primary root meristems. Compared with the sWT plants, the net K^+^ influx of *AvrPiz-t* transgenic seedlings was clearly lower through out of the 10-min interval measurement when supplied with 0.1 mM K^+^ (S4C Fig, upper panel). However, when the external K^+^ concentration was increased to 1.0 mM, no significant difference was detected (S4C Fig, bottom panel), which is consistent with the K^+^ content analysis. Taken together, these data indicate that AvrPiz-t can partially inhibit the K^+^ uptake in rice plants.

### *OsAKT1* is required for rice resistance against *M*. *oryzae*

**Fig 3:**
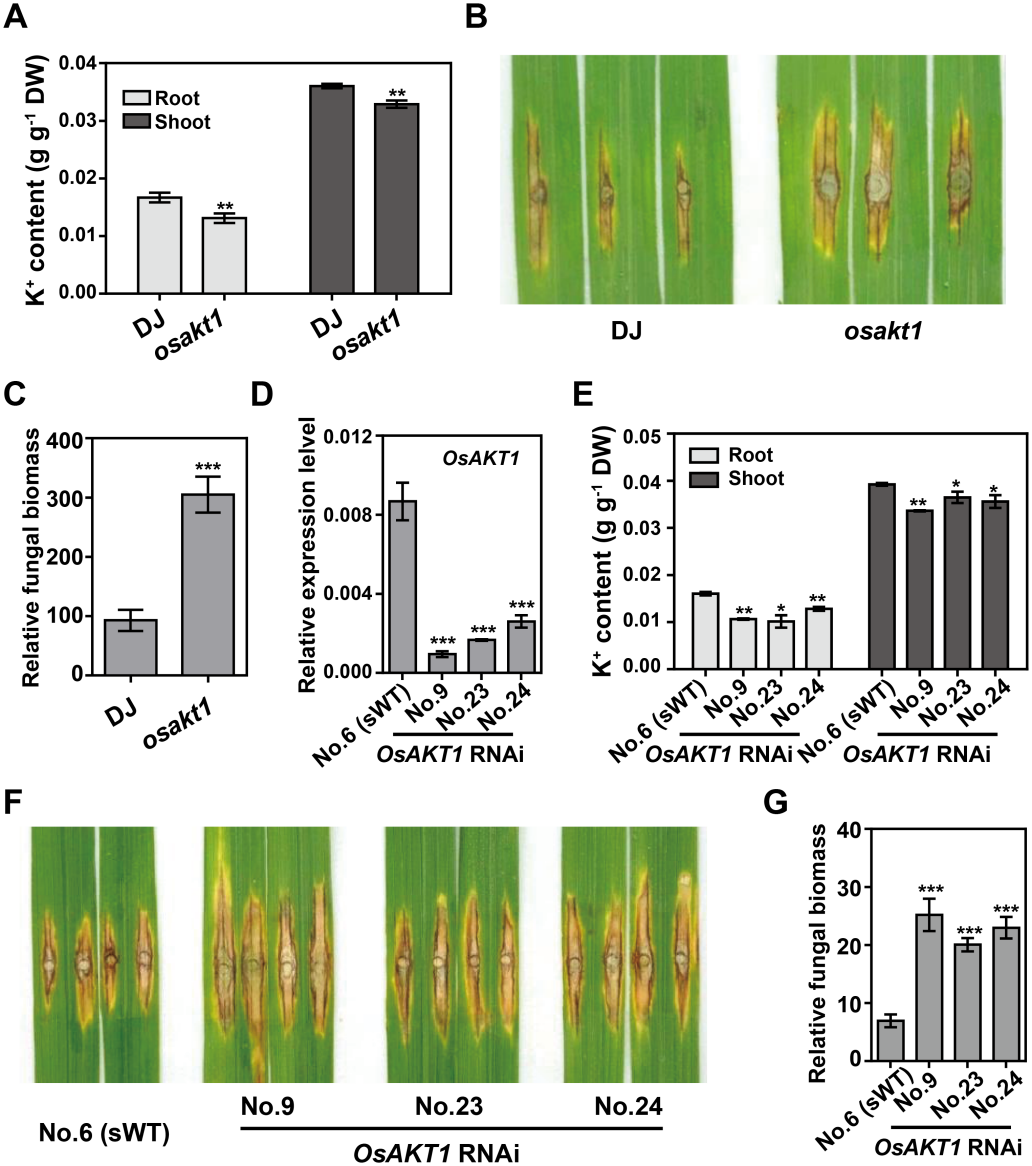
OsAKT1 is required for blast resistance and K^+^ uptake in rice. **(A)** K^+^ levels in Dongjin (DJ) and the *osakt1* mutant plants. Data represent the K^+^ weight in one gram of dry weight (DW) of plant tissues and are shown as means ± s.e.m. of three biological repeats. Student’s *t*-test (**P < 0.01). (**B, C**) Phenotypes of DJ and *osakt1* mutant inoculated with the compatible *M*. *oryzae* isolate RO1-1. The leaves were photographed 12 days after inoculation (**B**) and were subjected to DNA extraction to quantify the fungal growth by qPCR [2^[CT(*OsUbq*)-CT(*MoPot2*)]^] (**C**). The data are shown as means ± s.e.m. (*n* = 9). Student’s *t*-test (***P < 0.001). (**D, E**) *OsAKT1* expression (**D**) and K^+^ content (**E**) in *OsAKT1* RNAi and sWT (NPB background) plants. The data are shown as means ± s.e.m. of three biological repeats. Student’s *t*-test (*P < 0.05, **P < 0.01 and ***P < 0.001). (**F, G**) Phenotypes of *OsAKT1* RNAi lines inoculated with the compatible *M*. *oryzae* isolate RO1-1 (**F**) and the relative fungal growth, quantified by qPCR [2^[CT(*OsUbq*)-CT(*MoPot2*)]^/100] (**G**). The data are shown as means ± s.e.m. (*n* = 6). Student’s *t*-test (***P < 0.001).

OsAKT1 has been reported to play central roles in K^+^ uptake in rice [4]. To determine whether OsAKT1 contributes resistance to *M*. *oryzae*, we first analyzed its expression pattern in Nipponbare (NPB) plants that were infected by a compatible *M*. *oryzae* isolate. qRT-PCR analysis showed *OsAKT1* expression was highly induced at 24-hour post inoculation (hpi) and continued to increase until at least 120 hpi (S5A Fig). To assess the potential role of *OsAKT1* in rice blast resistance, we identified a T-DNA insertion knock-out mutant of *OsAKT1* in the Dongjin (DJ) background (S5B and S5C Fig). K^+^ content analysis showed that the *osakt1* mutant accumulated lower levels of K^+^ in roots and shoots compared with DJ plants (Fig 3A). We then punch-inoculated *osakt1* and DJ plants with a compatible *M*. *oryzae* isolate. Ten days after inoculation, the *osakt1* mutant plants showed larger disease lesions and more fungal biomass than DJ (Fig 3B and 3C).

To confirm the increased susceptibility phenotype of the *osakt1* mutant, we generated *OsAKT1* RNA interference (RNAi) plants in NPB background. T_1_ plants with a single T-DNA insertion and significantly reduced *OsAKT1* expression (Fig 3D) were selected for blast inoculation. Because *OsAKT1* has a homologue (*LOC_Os07g07910*) in the rice genome that shares 70% identity with *OsAKT1* [28], we tested the expression of this gene in the *OsAKT1* RNAi lines to determine the silencing specificity. The qRT-PCR results indicated that the expression of *LOC_Os07g07910* was not significantly affected in the *OsAKT1* RNAi lines, indicating the specificity of the RNAi fragment (S6 Fig). Similar to the *osakt1* mutant, the *OsAKT1* RNAi lines showed a significant decrease in K^+^ content in roots and shoots (Fig 3E), as well as enhanced susceptibility to *M*. *oryzae*, with increased fungal biomass in the lesion area compared with the sWT plants (Fig 3F and 3G). Together, these results demonstrate that *OsAKT1* plays a positive role in rice immunity to *M*. *oryzae*, likely by modulating K^+^ absorption.

### AvrPiz-t competes with OsCIPK23 for binding to OsAKT1

AvrPiz-t can promote the degradation of its target proteins APIP6 and APIP10 [24, 25]. To investigate whether AvrPiz-t affects OsAKT1 protein stability, we co-expressed AvrPiz-t with OsAKT1 intracellular fragments or the full-length OsAKT1 in rice protoplasts. However, immunoblot analysis showed that AvrPiz-t did not obviously affect the stability of OsAKT1-C, OsAKT1-C1 and the full-length OsAKT1 proteins (S7A–S7C Fig), indicating a different regulation mechanism from APIP6 and APIP10.

**Fig 4:**
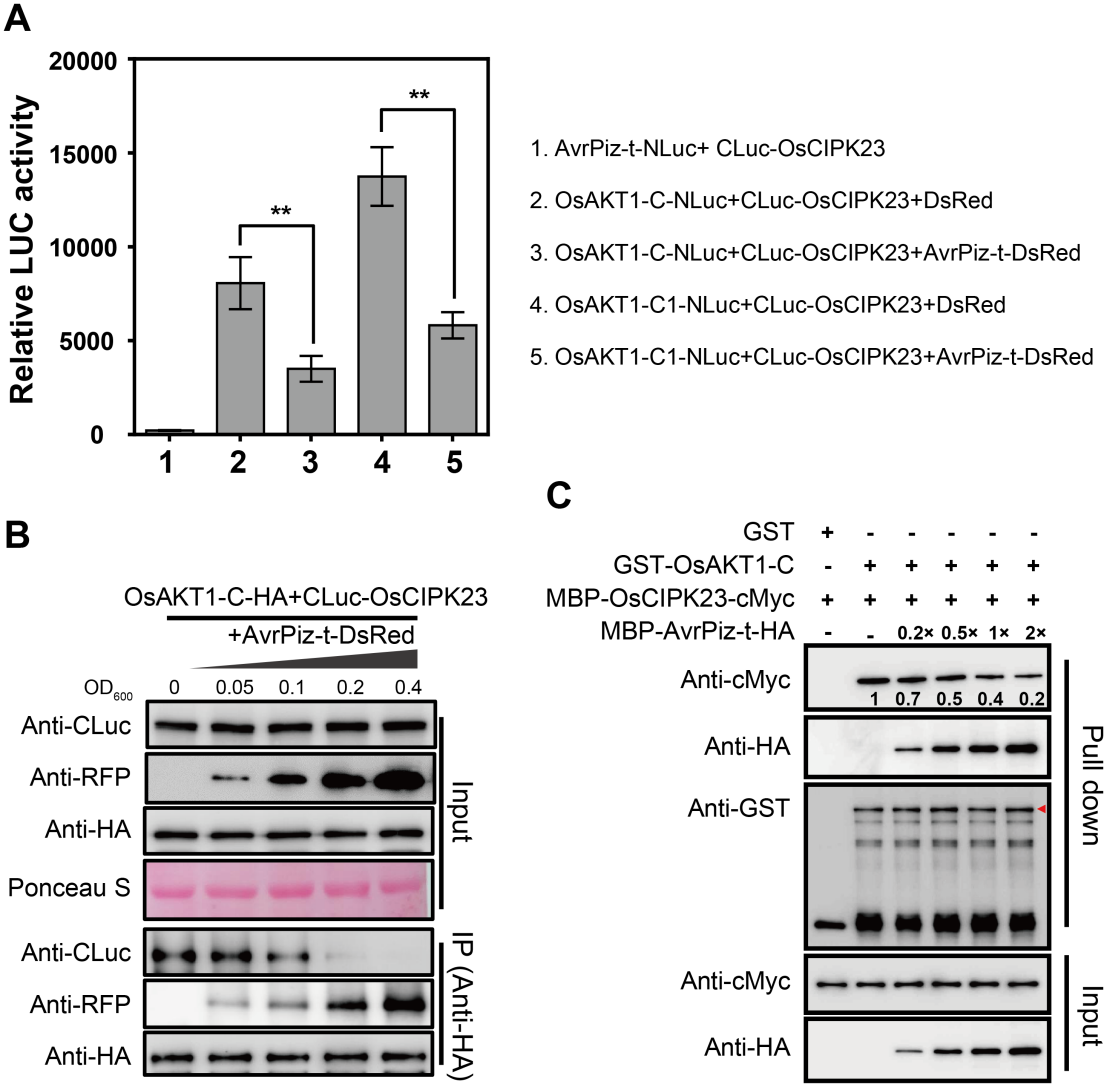
AvrPiz-t competes with OsCIPK23 for binding to OsAKT1. (**A**) AvrPiz-t attenuates the interaction between OsCIPK23 and OsAKT1 in *N*. *benthamiana*. The indicated NLuc and CLuc constructs were transiently co-expressed with DsRed or AvrPiz-t-DsRed in *N*. *benthamiana* leaves for the luciferase complementation assay. Data are shown as means ± s.e.m. of three biological repeats. Student’s *t*-test (**P < 0.01). (**B**) AvrPiz-t decreases the association of OsAKT1 and OsCIPK23 in a competitive Co-IP assay. OsAKT1-C-HA and CLuc-OsCIPK23 were co-expressed with increasing amount of AvrPiz-t-DsRed. *Agrobacteria* carrying different constructs were adjusted to optical density 600 (OD_600_) = 0.2 (*OsAKT1-C-HA*), OD_600_ = 0.1 (*CLuc-OsCIPK23*) and OD_600_ = 0–0.4 (*AvrPiz-t-DsRed*). The expressed proteins were immunoprecipitated with anti-HA antibody. (**C**) The *in vitro* GST pull-down assay shows the dosage-dependent effect of AvrPiz-t on the OsCIPK23-OsAKT1 interaction. The MBP-OsCIPK23-cMyc proteins were incubated with GST or GST-OsAKT1-C along with increasing amounts of MBP-AvrPiz-t-HA in a pull-down assay. The amounts of proteins were detected by immunoblot analysis. The relative protein levels of retrieved MBP-OsCIPK23-cMyc were analyzed with Image J. Red arrow indicates the GST-OsAKT1-C protein. These experiments were repeated three times with similar results.

OsAKT1-mediated K^+^ uptake is regulated by the OsCIPK23 complex [4]. Since a previous study showed that CBL10 can compete with CIPK23 for binding to AKT1 and thus negatively regulates AKT1 activity in *Arabidopsis* [31], we attempted to determine whether AvrPiz-t interferes with the association of OsAKT1 with OsCIPK23 in a similar manner. We first confirmed the interaction between OsAKT1 and OsCIPK23 by luciferase complementation and Co-IP assays in *N*. *benthamiana*. The assay showed that both OsAKT1-C and OsAKT1-C1 interacted with OsCIPK23 while AvrPiz-t did not (S8A and S8B Fig). To ascertain whether AvrPiz-t interferes with the OsAKT1-OsCIPK23 interaction, we included AvrPiz-t-DsRed in the luciferase complementation assay. Compared with the DsRed control combination, the relative luciferase activity significantly decreased when AvrPiz-t-DsRed was co-expressed with OsAKT1-C or -C1 and OsCIPK23 (Fig 4A, lanes 2 to 5). This result was further confirmed in a competitive Co-IP assay. We transiently co-expressed a same amount of OsAKT1-C-HA and CLuc-OsCIPK23 in *N*. *benthamiana* leaves with an increasing amount of AvrPiz-t-DsRed by adding different concentrations of *Agrobacteria* carrying *AvrPiz-t-DsRed*. Immunoblot analysis showed that, as AvrPiz-t levels were increased (Fig 4B, second panel from the bottom), the immunoprecipitated OsCIPK23 protein levels were decreased significantly (Fig 4B, third panel from the bottom). Next, we performed a pull-down assay to establish whether AvrPiz-t interferes with the OsCIPK23-OsAKT1 association *in vitro*. The results showed that GST-OsAKT1-C bound to both MBP-AvrPiz-t-HA and MBP-OsCIPK23-cMyc at the same time; however, with increasing amounts of MBP-AvrPiz-t-HA (Fig 4C, panels 2 and 5), the enrichment of MBP-OsCIPK23-cMyc gradually decreased to 20% compared with the control (Fig 4C, panel 1, lanes 2 to 6). As the controls, GST-OsAKT1-C did not pull down MBP-AvrPii-HA and increasing the amount of MBP-AvrPii-HA protein did not obviously affect the protein levels of retrieved MBP-OsCIPK23-cMyc compared with MBP-AvrPiz-t-HA (S9 Fig). These results demonstrate that AvrPiz-t specifically interferes with the OsAKT1-OsCIPK23 association both *in vivo* and *in vitro*.

### Loss function of *OsCIPK23* impairs resistance to *M*. *oryzae*

**Fig 5:**
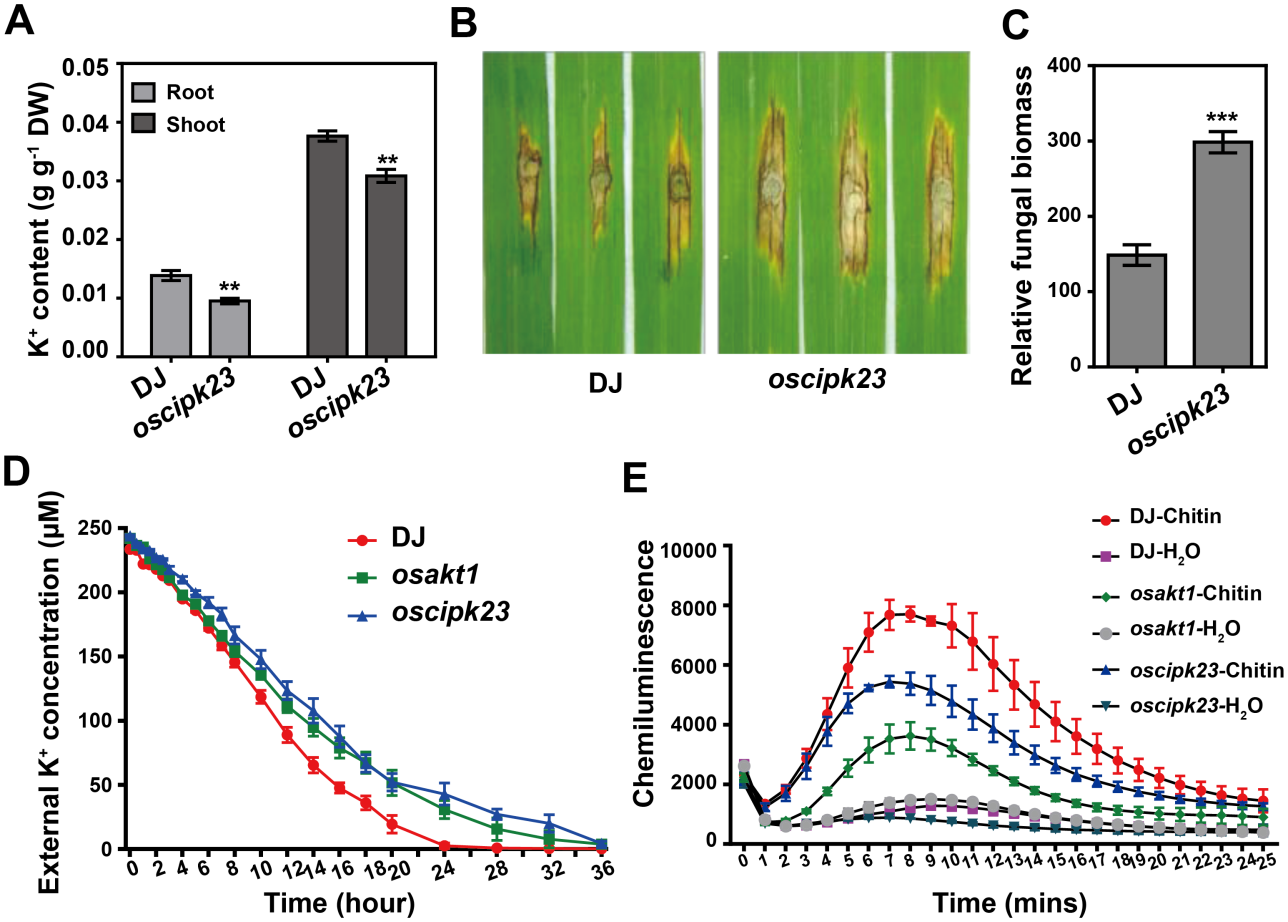
Loss functions of *OsCIPK23* impairs blast disease resistance and K^+^ absorption in rice. (**A**) K^+^ content in DJ and *oscipk23* mutant plants. Data are shown as means ± s.e.m. of three biological repeats. Student’s *t*-test (**P < 0.01). (**B, C**) Phenotype of *oscipk23* mutant inoculated with the compatible *M*. *oryzae* isolate RO1-1 (**B**), and the relative fungal biomass was determined by qPCR [2^[CT(*OsUbq*)-CT(*MoPot2*)]^] (**C**). Data are shown as means ± s.e.m. (*n* = 9). Student’s *t*-test (***P < 0.001). (**D**) K^+^ depletion assay was performed to show the K^+^ uptake ability of DJ, *osakt1* and *oscipk23* plants. Data are shown as means ± s.e.m. of three biological replicates. (**E**) Chitin-induced ROS accumulation in DJ, *osakt1* and *oscipk23* plants were determined with luminol-chemiluminescence assay with H_2_O treatment as negative control. Data are shown as means ± s.e.m. of three biological replicates.

The interference of AvrPiz-t with the OsAKT1-OsCIPK23 interaction prompted us to investigate the function of *OsCIPK23* in rice immunity. We measured the expression of *OsCIPK23* during *M*. *oryzae* infection and found that its transcription increased at 72 hpi (S10A Fig). Then we identified a T-DNA insertion mutant of *OsCIPK23* in the DJ background (S10B and S10C Fig). The K^+^ content of *oscipk23* mutant roots and shoots was much lower than that in DJ plants (Fig 5A), which is similar to *OsCIPK23* RNAi plants [4]. A punch inoculation experiment showed that *oscipk23* mutant plants were more susceptible to a compatible *M*. *oryzae* isolate (Fig 5B), showing increased fungal growth in the lesion area compared with the DJ plants (Fig 5C).

To confirm the phenotype of *oscipk23*, we generated gene-editing plants of *OsCIPK23* in the NPB background with clustered regularly interspaced short palindromic repeat (CRISPR) and CRISPR associated protein 9 (Cas9) system [32, 33]. We designed two single-guide RNAs (sgRNAs) in one construct which targets two different sites followed with protospacer adjacent motifs (PAMs) in the first exon of *OsCIPK23* (S11A Fig). Based on PCR and sequencing analysis, we selected three homozygous lines with different mutation types to test their resistance to *M*. *oryzae* (S11B Fig). Consistent with the T-DNA insertion mutant, the punch inoculation showed all these three lines displayed reduced resistance to the compatible *M*. *oryzae* isolate (S11C and S11D Fig).

Since AvrPiz-t weakens both K^+^ uptake (S4A Fig) and ROS accumulation in rice [24], we performed similar K^+^ depletion and PAMP-induced ROS detection assays using *osakt1* and *oscipk23* mutant plants. The results indicated that both *osakt1* and *oscipk23* mutant plants showed reduced K^+^ uptake and ROS burst after treatment with the PAMP chitin compared with DJ plants (Fig 5D and 5E). Taken together, these results suggest that, similar to OsAKT1, OsCIPK23 positively regulates immunity to *M*. *oryzae* and K^+^ absorption in rice.

### High levels of K^+^ enhance *M*. *oryzae* resistance in rice

**Fig 6:**
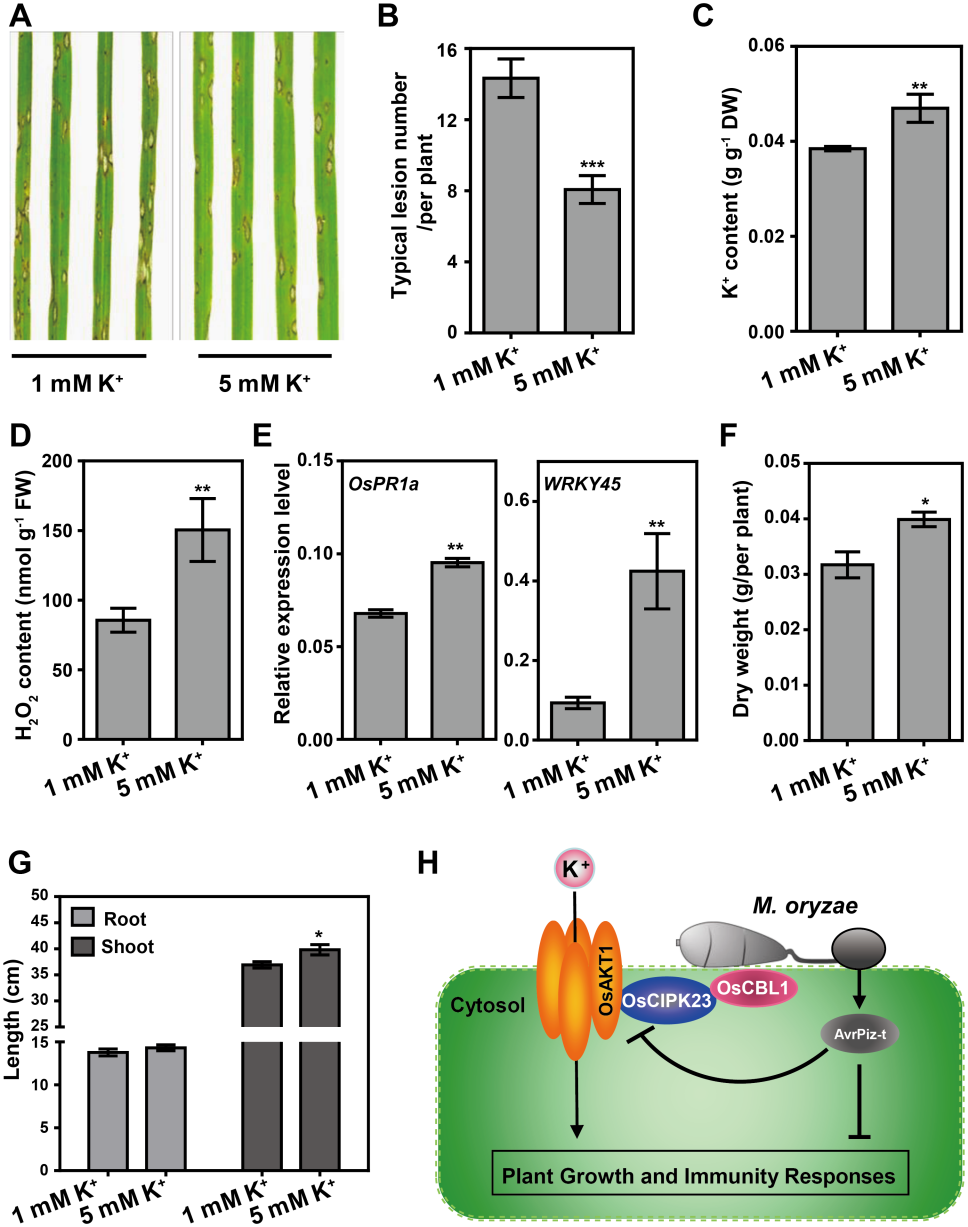
High levels of K^+^ enhance rice blast disease resistance with increased plant biomass. (**A**) Phenotypes of rice NPB plants cultivated in hydroponic solutions containing 1 mM or 5 mM K^+^ challenged with the compatible *M*. *oryzae* isolate RO1-1. (**B**) The disease symptoms in (**A**) were evaluated by counting the number of disease lesions on leaves. Data are represented as means ± s.e.m. (*n* = 15). Student’s *t*-test (***P < 0.001). (**C-E**) Detection of K^+^ content (**C**), basal H_2_O_2_ level (**D**) and relative expression level of defense-related marker genes (**E**) of plants cultivated in 1 mM or 5 mM K^+^ hydroponic solution. Data are represented as means ± s.e.m. (*n* = 3) Student’s *t*-test (**P < 0.01). (**F, G**) Higher K^+^ level increases plant biomass. Dry weight (**F**) as well as root and shoot length (**G**) are measured in NPB plants cultivated in 1 mM and 5 mM K^+^ solution. Data are shown as means ± s.e.m. (*n* = 6) Student’s *t*-test (*P < 0.05). (**H**) A working model to illustrate the relationship between AvrPiz-t and OsAKT1-mediated K^+^ uptake in rice. In normal conditions, the OsCBL1-OsCIPK23-OsAKT1 complex modulate K^+^ signal transduction for plant growth and development, and disease resistance. To overcome this barrier, pathogens have evolved effectors (like AvrPiz-t) to suppress the activity of OsAKT1 and/or destroy the OsAKT1-OsCIPK23 complex to subvert K^+^ signal transduction to facilitate their infection and colonization.

As shown above, loss function of *OsAKT1* and *OsCIPK23* has decreased rice resistance to *M*. *oryzae*, and ectopic expression of *AvrPiz-t* has impaired the K^+^ uptake and reduced blast resistance, indicating that K^+^ has a positive role in rice immunity against *M*. *oryzae*. To further investigate the effect of K^+^ on rice immunity, we used a compatible *M*. *oryzae* isolate to inoculate NPB seedlings which were grown in 1 mM or 5 mM K^+^ solution to mimic normal and high K^+^ levels [5, 34], respectively. Five days after inoculation, we observed a significant decrease in disease symptoms on the rice plants cultivated in 5 mM K^+^ compared with those grown in 1 mM K^+^ (Fig 6A and 6B). We measured the K^+^ content in the whole plants and found that the K^+^ content is ~ 20% higher in the 5 mM K^+^ plants than in the 1 mM K^+^ plants (Fig 6C). In addition, the basal H_2_O_2_ levels as well as two defense-related genes, *OsPR1a* and *WRKY45*, were elevated in the plants cultivated in the higher K^+^ conditions (Fig 6D and 6E). Furthermore, we observed increased dry weight and shoot lengths in the plants cultivated in the 5 mM K^+^ solution (Fig 6F and 6G), suggesting a positive function of K^+^ in regulating plant development.

To test the direct effect of K^+^ on *M*. *oryzae* growth, we cultured two isolates on the complete medium that contained additional 40, 80, 120, 150 and 200 mM K^+^ according to the estimated K^+^ concentration in plant cytosol (ranged from 100–200 mM [35]). The colony diameters were measured 14 days after inoculation. The results showed that with increasing of K^+^ concentration, the fungus growth was gradually inhibited (S12A and S12B Fig). Taken together, these results indicate that relative high external K^+^ concentration promotes plant growth and blast resistance; however, the enriched K^+^ in plant cells may has a negative effect on fungal growth.

## Discussion

AKT1 is one of the major K^+^ uptake components in *Arabidopsis* [36]. The electrophysiological activity of AKT1 is positively regulated by CBL1/9-CIPK23 complexes [36], but suppressed by the 2C-type protein phosphatase AIP1 [37], the Shaker family α-subunit AtKC1[38], and CBL10 [31]. The *Arabidopsis* AKT1 homolog in rice, OsAKT1, has also been characterized as an important potassium channel for K^+^ absorption [4]. However, the *AKT1* gene in both plants has not previously been associated with immunity to pathogens. In this study, we show that the *M*. *oryzae* effector AvrPiz-t interacts with OsAKT1 and suppresses OsAKT1-mediated inward K^+^ currents (Figs 1 and 2), thereby likely interfering with the K^+^ signaling pathway. Genetic analysis showed that loss of *OsAKT1* function led to reduced K^+^ content, K^+^ uptake and enhanced susceptibility to *M*. *oryzae* (Figs 3 and 5D). Consistently, we observed a decrease of K^+^ uptake and K^+^ content in the *AvrPiz-t* transgenic plants at lower K^+^ levels (S4 Fig). We also found that elevated K^+^ concentrations in rice tissues can enhance resistance against *M*. *oryzae* (Fig 6A–6C) and high K^+^ concentrations in culture medium can inhibit *M*. *oryzae* growth (S12 Fig), suggesting that rice may absorb more K^+^ for immune activations and fungal growth inhibition during *M*. *oryzae* infection. It has been reported that the bacterial pathogen *Xanthomonas oryzae* overcomes rice defenses by regulating the redistribution of the micromutrient copper through activation of XA13, COPT1 and COPT5 [39]. Our study has revealed a new strategy by *M*. *oryzae* to interfere the OsAKT1 complex to modulate the uptake of the macronutrient K^+^ in favor of fungal pathogenesis in rice. However, it is still not fully clear how high K^+^ level in the host enhances immunity against *M*. *oryzae*.

In *Arabidopsis*, CBL1/9 interacts with CIPK23 and recruits it to the plasma membrane, where CIPK23 phosphorylates and activates AKT1 to mediate K^+^ absorption [36, 40]. As CBL proteins act as Ca^2+^ sensors in plant cells, the activation of AKT1 by CBL1/CBL9-CIPK23 is reported to be in a Ca^2+^-dependent manner [40]. As a second messenger, the cytosolic free Ca^2+^ concentration ([Ca^2+^]_cyt_) is elevated upon PAMP (e.g. flg22, elf18 and chitin) treatments [3, 41, 42]. Although the elevated [Ca^2+^]_cyt_ is accompanied with rapid membrane depolarization and K^+^ efflux at the earlier stage [43, 44], the activation of CBL proteins by accumulated [Ca^2+^]_cyt_ may induce K^+^ acquisition at the late stage. The observation of induction of *OsAKT1* and *OsCIPK23* at the later stage of *M*. *oryzae* infection may further strengthen this hypothesis (S5A and S10A Figs). CIPK23 is not only a master regulator of AKT1, but also regulates the activity of the K^+^ transporter HAK5, the NO_3_^-^ sensor CHL1 and the NH_4_^+^ transporters AMT1;1 and AMT1;2 [45–47]. Thus, CBL1/9-CIPK23 cascade plays key roles in regulating the K^+^, NO_3_^-^ and NH_4_^+^ homeostasis in *Arabidopsis* [47]. The activation of CIPKs relies on the direct interaction of the self-inhibitory NAF motif with a particular CBL protein [48–51]. The rice OsAKT1 protein acting alone can mediate inward K^+^ currents in HEK293 cells, while OsCBL1 and OsCIPK23 further enhance the strength of the K^+^ currents [4]. Physiological analyses have demonstrated that both OsAKT1 and OsCIPK23 positively regulate K^+^ absorption [4], and here we show that AvrPiz-t competes with OsCIPK23 for binding to OsAKT1 (Fig 4). This may affect the stability of the OsAKT1-OsCIPK23 complex and decrease OsAKT1 activity. Although it has been reported that activation of AKT1 in *Arabidopsis* depends on the phosphorylation of CIPK23 [36, 40], we were not able to demonstrate that OsCIPK23 has kinase activity *in vitro*, even when incubated with OsCBL1 or deleted the self-inhibition NAF motif as indicated in *Arabidopsis* CIPK23 [36]. We speculate that OsCIPK23 kinase activity may require another unknown protein except OsCBL1. Consistent with the AvrPiz-t competition effect, the *oscipk23* mutant showed significantly reduced K^+^ levels and enhanced susceptibility to *M*. *oryzae* (Fig 5A–5C). Moreover, the *osakt1* and *oscipk23* mutants showed impaired K^+^ uptake and ROS accumulation (Fig 5D and 5E), which provides further evidence for the importance of K^+^ in enhancing resistance to *M*. *oryzae* in rice. PexRD54, an effector protein secreted by the oomycete pathogen *Phytophthora infestans*, also utilizes a competition mechanism to subvert host defense [12]. PexRD54 directly binds to ATG8CL and competes with the host cargo receptor Joka2 that causes Joka2 out of the ATG8CL autophagosomes, thereby promoting disease susceptibility [12]. PsAvh23, another effector from the soybean pathogen *Phytophthora sojae*, attacks ADA2 subunit of histone acetyltransferase SAGA to block the association of ADA2 with the catalytic subunit of GCN5, and thus to suppress H3K9 acetylation and increase plant susceptibility [52]. Those examples suggest that pathogen effectors may interfere the function of host protein complex by competitive binding.

In conclusion, our study reveals a novel function of AvrPiz-t, as well as the role of OsAKT1-OsCIPK23-mediated K^+^ signaling in rice innate immunity. We provide evidence of an intimate connection between plant nutrition status and disease resistance and propose a working model depicting the mechanism by which AvrPiz-t promotes the infection of the blast fungus via perturbing the function of the K^+^-associated OsAKT1-OsCIPK23 complex in rice (Fig 6H).

## Materials and methods

### Plant materials and growth conditions

For hydroponic cultivation, the rice seeds were sown on wet filter paper and pre-germinated in an incubator for 3 days. The germinated seeds were transferred to nutrient solution containing 1 mM or 5 mM K^+^. The solution was replaced every 3 days. For the transgenic plants and T-DNA insertion mutants, seeds were germinated on half-strength Murashige and Skoog (MS) medium containing 50 mg/L hygromycin for 10 days and then transferred to soil. Seedlings were kept in a growth chamber at 26°C and 70% relative humidity with a 12-h light/dark photoperiod.

### Blast fungus inoculation and disease symptom evaluation

The *M*. *oryzae* isolates RO1-1 and RB22 were cultivated on oat meal medium under weak light for 2 weeks to generate spores. For spray inoculation, three-week-old seedlings were sprayed with 1–1.5×10^5^/mL spores of *M*. *oryzae* as previously described [53]. 5–7 days after inoculation, the typical susceptible lesion numbers in each seedling were counted to evaluate the infection level.

Four-six weeks old rice plants were tested with the punch inoculation method as previously described [24]. Different from spay inoculation, the concentration of spores is higher for punch inoculation (about 5×10^5^/mL). The rice leaves were lightly punched with a mouse ear clip and a 10 μL volume of spore suspension was dropped on the punched sites of leaves. Then the spore suspension was hold by sealing with Scotch tape on both sides. Twelve days after inoculation, the inoculated leaves were photographed and the relative fungal biomass was calculated to determine the fungal growth in leaves. The calculation of the relative fungal biomass was performed as described before [24]. Briefly, 4 cm of rice leaf with lesion was cut for DNA extraction with classical cetyltrimethyl ammonium bromide (CTAB) extraction protocol. Relative fungal biomass was measured with DNA-based quantitative PCR (qPCR) using the threshold cycle value (CT) of *M*. *oryzae MoPot2* gene against the CT value of rice genomic *Ubiquitin* (*OsUbq*) gene according to the formula 2^[CT(*OsUbq*)-CT(*MoPot2*)]^. The qPCR was performed with 2×SYBR Green Mix (GeneStar) on ABI Prism 7500 PCR instrument. Primers used for analysis were listed in S1 Table.

### Yeast two-hybrid (Y2H) and GST pull-down analysis

The Y2H and GST pull-down assays were performed as previously described [27] with slight modifications. Briefly, the ProQuest Two-Hybrid System (Invitrogen) was used for the Y2H experiments. The *AvrPiz-t* coding region without the predicted signal peptide sequence [24] was cloned in-frame into the pDBleu vector as the bait. The sequences encoding the *OsAKT1* intracellular C terminal fragments were cloned into the pPC86 vector as the prey. After co-transformation of yeast (*Saccharomyces cerevisiae* strain Mav203) and screening on SD/-Leu-Trp plates, positive clones were selected to grow on SD/-Leu-Trp-His medium with different concentration of 3-Amino-1,2,4-Triazole (3AT), or tested with β-galactosidase (X-gal).

For the GST pull-down assay, ~10 μg GST fusion proteins were mixed with ~10 μg or the indicated amount of MBP fusion proteins. The mixtures were incubated at room temperature for 1 h with gentle shaking, and then 20 μL pre-rinsed glutathione sepharose beads (GE Healthcare) was added, followed by incubation at room temperature for another 1 h. The beads were then washed 5–7 times with 1× TBST buffer. Finally, 1× SDS sample-loading buffer was added to the beads, and the mixture boiled for 5 min, prior to SDS-PAGE analysis.

### *In vivo* luciferase complementation and Co-IP assay in *N*. *benthamiana*

The luciferase complementation assay was conducted as previously described [54]. *Agrobacterium tumefaciens* (strain EHA105) containing the desired constructs was used to infiltrate *N*. *benthamiana* leaves after adjusting the concentration of bacterial solution with MES buffer (10 mM MgCl_2_, 10 mM MES, pH 5.6) to optical density 600 (OD_600_) of 0.5. At 36 h after infiltration, leaf discs were taken and incubated with 150 ng/ml D-luciferin potassium in a 96-well plate, and the relative LUC activity was detected with a GLOMAX 96 microplate luminometer (Promega).

For the Co-IP assays, proteins were extracted from infiltrated leaf tissues with native buffer (50 mM Tris-MES, pH 8.0, 0.5 M sucrose, 1 mM MgCl_2_, 10 mM EDTA, 5 mM DTT, and protease inhibitor cocktail) and were subjected to anti-HA immunoprecipitation. After incubation with HA antibody (Roche) for 4 h at 4°C, 20 μL pre-rinsed Protein G beads (Millipore) was added to the protein-antibody mixtures and incubated for another 3 h. The beads were then washed 3–5 times with 1× TBST buffer. 1× SDS loading buffer was added to the samples and boiled for 5 min to elute the proteins prior to SDS-PAGE and immunoblot analysis.

### Patch-clamp analysis of HEK293 cells

The patch-clamping experiments were performed as described previously [4]. Briefly, HEK293 cells (human embryonic kidney cell line 293) were purchased from ATCC (American Type Culture Collection) and pre-cultured in Dulbecco’s modified eagle medium with 4.5 g/L glucose and 10% fetal calf serum for 24 h at 37°C with 5% CO_2_. *OsAKT1* full length CDS was cloned in-frame in the pEGFP-N1 vector (Clontech). *DsRed* was used to monitor the transfection efficiency and was added to the pBudCE4.1 vector (Invitrogen) to allow expression driven by the EF-1α promoter, while *AvrPiz-t* or *AvrPii* variants were cloned into another site in the vector under control of the CMV promoter. The constructs pEGFP-N1-OsAKT1 and pBudCE4.1-DsRed-AvrPiz-t/AvrPii were co-transfected into HEK293 cells and the transfected cells were collected by centrifugation at 160 *g* for 5 min. The cells with both GFP and RFP fluorescence were selected for whole-cell recording, which was conducted at 20°C in dim light with an Axopatch 200B amplifier (Axon Instruments). The pipette solution and batch solution were as previously described [4]. Primers used for vector constructions were listed in S1 Table.

### K^+^ content measurement and K^+^ depletion assay

The shoots and roots of three-week-old plants were collected, rinsed with deionized water and dried at 65°C to a constant weight (at least 3 days). The dry samples were weighed and then incinerated in a muffle furnace at 300°C for 1 h and 575°C for 7 h, as previously described [4]. After incineration, the ashes were dissolved in 0.1 N hydrochloric acid and diluted with water to a suitable K^+^ concentration (~100 mM) based on the K^+^ content of rice (~3%). The K^+^ concentrations were determined by microwave plasma emission spectrometry (Agilent 4100 MP-AES).

The K^+^ depletion assay was performed as previously described [4]. Rice seeds were germinated on half-strength MS medium at 28°C under full light. Seven days after germination, 7 seedlings (fresh weight ~0.8g) with the endosperm removed were pretreated with starvation solution (0.2 mM CaSO_4_, 5 mM MES, pH 5.75) for 18 h then transferred to depletion solution (0.25mM KNO_3_, 0.2 mM CaSO_4_, 5 mM MES, pH 5.75). The treatments were conducted at 28°C under full light on a shaking table and samples were collected at different time points.

### Gene expression analysis

Rice leaf samples were collected from three-week-old seedlings for DNA or RNA isolation. DNA was isolated with CTAB buffer (2% CTAB, 100 mM Tris-HCl, 20 mM EDTA, 1.4 M NaCl, 0.1% 2-mercaptoethanol). Total RNA was isolated with Trizol regent (Invitrogen) according to the manufacturer’s instruction. First strand cDNA was synthesized with reverse transcriptase (Promega) after digestion of total RNA with DNase (TransGen). Genomic PCR and semi-quantitative PCR were performed with 2×TSINGKE Master Mix (TSINGKE). qPCR was performed with 2×SYBR Green Mix (GeneStar) on ABI Prism 7500 PCR instrument. Gene expression levels were calculated with the data from three technical repeats. Primers used for analysis were listed in S1 Table.

### Net K^+^ flux analysis in rice root meristem with non-invasive micro-test technology (NMT)

Rice seeds were germinated on half-strength MS medium at 28°C under 12 h dark and 12 h light for 10 days. Seedlings were removed endosperm and placed in starvation solution for 2 days. The net K^+^ flux was measured at the Xuyue (Beijing) Biofunction Institute by using NMT (NMT100 series; YoungerUSA LLC, Amherst, MA01002, USA) and imFluxes V2.0 software (YoungerUSA LLC, Amherst, MA01002, USA). The roots of seedlings were first equilibrated in measuring buffer (0.1 mM CaCl_2_ and 0.2 mM MES, PH 6.0) and then transferred to a measuring chamber with measuring buffer containing either 0.1 mM or 1 mM K^+^. The ion-selective electrodes were calibrated with measuring buffer containing 0.05, 0.1, and 0.5 mM K^+^ before measurement. Net K^+^ fluxes were measured for 10 min under experimental conditions. At least 6 individual plants were measured in an independent experiment.

### H_2_O_2_ content measurement and ROS kinetic analysis

To measure H_2_O_2_ levels in seedlings, we used a H_2_O_2_ detection ELISA kit (Kmaels DRE-P9104c). Seedlings (fresh weight ~200mg) were homogenized using a blender in 2 mL acetone and centrifuged at 10,000 *g* for 10 min. The supernatant was assayed for H_2_O_2_ concentration. The standard samples and testing samples were added to the ELISA plate according to the manufacturer’s instructions. After the reaction, the optical density was detected at 450 nm (OD_450_) using a microtiter plate reader within 15 min. A standard curve was generated by plotting the average OD_450_ of the standard samples.

For the ROS kinetic analysis, leaf disks were cut from 6-week-old plants and preincubated in sterile distilled water for about 10 h. Three leaf disks were transferred to a 1.5 mL microcentrifuge tube (Axygen) containing 100 μL of luminol (Bio-Rad Immun-Star horseradish peroxidase substrate), 1 μL of horseradish peroxidase and also 8 nM chitin (hexa-N-acetyl-chitohexaose) or water. Luminescence was then immediately measured using a Glomax 20/20 luminometer. Three biological replicates were assayed for each sample.

### Statistics

Statistical analyses were performed using GraphPad Prism 7.0 software. Descriptions of the tests used are introduced in figure legends.

### Accession numbers

Sequence data of rice genes can be found in the Rice Genome Annotation Project under following accession numbers: LOC_Os01g45990 for *OsAKT1* and LOC_Os07g05620 for *OsCIPK23*. The gene sequences of *M*. *oryzae* can be found in the Gene Bank database with the following accession codes: EU837058 for *AvrPiz-t* and AB498874 for *AvrPii*.

## Supporting information

S1 Fig Determination of the interaction region in OsAKT1 and AvrPiz-t. (**A**) The schematic illustration of the fragments of OsAKT1 used in yeast two-hybrid (Y2H) analysis. (**B**) Y2H analysis of the interaction domain of OsAKT1 with AvrPiz-t. The truncated OsAKT1 intracellular C terminal regions (**A**) were co-transfected with AvrPiz-t into yeast cells and screened with SD/-Leu-Trp-His (SD/-L-W-H) medium containing 35 mM 3-amino-1,2,4-triazole (3AT) and X-gal solution. (**C**) The interaction between AvrPiz-t(C70A) and OsAKT1-C1. TM, transmembrane domains; cNMP, cyclic nucleotide-monophosphate binding domain; ANK, ankyrin repeats.
(TIF)

S2 Fig The constructs used in the electrophysiology experiments and protein detection by western blot analysis. (**A**) Schematic diagram showing the constructs used in the electrophysiology experiments. (**B**) Western blot analysis of the expression of proteins in the HEK293 cells. Proteins were extracted from the HEK293 cells transfected with indicated constructs and detected by western blot analysis with Anti-GFP and Anti-RFP antibodies, respectively.
(TIF)

S3 Fig The effect of AvrPii to OsAKT1-mediated inward K^+^ currents. (**A**) Y2H analysis of the interaction between OsAKT1 and AvrPii. The interactions of OsAKT1 intracellular C terminal fragments and AvrPii were tested by X-gal solution and SD/-L-W-H medium containing different concentrations of 3AT. Lower concentrations of 3AT were designed to determine possibly weak interaction. AvrPiz-t was used as the positive control. (**B**) Patch-clamp whole-cell recordings of inward K^+^ currents in HEK293 cells expressing OsAKT1-GFP, AvrPii+DsRed+OsAKT1-GFP, FLAvrPii+DsRed+OsAKT1-GFP. The voltage protocols, as well as time and current scale bars for the recordings, are shown. (**C**) The I-V relationship of the steady state whole-cell inward K^+^ currents in HEK293 cells. The data are derived from the recordings as shown in (**B**) and presented as means ± s.e.m. (OsAKT1-GFP, *n* = 36; AvrPii+DsRed+OsAKT1-GFP, *n* = 23; FLAvrPii+DsRed+OsAKT1-GFP, *n* = 19).
(TIF)

S4 Fig K^+^ uptake is impaired in rice seedlings expressing *AvrPiz-t*. (**A**) Comparison of K^+^ uptake ability of sWT and *AvrPiz-t* transgenic plants with K^+^-depletion assay. Data are shown as means ± s.e.m. (*n* = 3). (**B**) K^+^ levels in the shoots of sWT and *AvrPiz-t* transgenic rice seedlings cultivated in 0.1 mM or 1 mM K^+^ hydroponic solution for 3 weeks. Data are shown as means ± s.e.m. (*n* = 3). (**C**) Comparison of K^+^ fluxes in the primary root meristem of sWT and *AvrPiz-t* transgenic plants. The left panels indicate the net K^+^ fluxes supplied with 0.1 mM or 1mM K^+^ for 10 min. Data are means ± s.e.m. from 5 individual plants. The mean K^+^ fluxes over the 10 min were shown in the right column diagrams. Data are shown as means ± s.e.m. (*n* = 10). Student’s *t*-test (**P < 0.01). ns, no significant difference.
(TIF)

S5 Fig *OsAKT1* expression pattern during *M*. *oryzae* infection and identification of the *osakt1* mutant. (**A**) Nipponbare (NPB) plants were sprayed with the compatible *M*. *oryzae* isolate RB22 or mock (without pathogen). The shoots samples were collected at indicated time points and subjected to RNA extraction. *OsAKT1* specific primers are used to do the qRT-PCR. Data are shown as means ± s.e.m. (*n* = 3). (**B**) The schematic illustration of the structure of the *OsAKT1* gene and the T-DNA insertion site of the *osakt1* mutant. The black boxes indicate exons and the lines represent introns. Sites of primers used for identification of the mutant are labeled with arrows. (**C**) Genomic and transcriptional identification of the *osakt1* mutant. The *Ubiquitin* (*Ubq*) gene was used as internal control.
(TIF)

S6 Fig The expression level of *LOC_Os07g07910* in the *OsAKT1* RNAi lines. Relative gene expression of *LOC_Os07g07910* was measured by qRT-PCR in the leaves of *OsAKT1* RNAi lines. Values represent means ± s.e.m. (*n* = 3). ns, no significant difference.
(TIF)

S7 Fig AvrPiz-t doesn’t affect the stability of OsAKT1 in rice protoplasts. ~5 μg *OsAKT1-C-HA* (**A**), *OsAKT1-C1-HA* (**B**) or *OsAKT1-HA* (**C**) construct was co-transfected with ~5 μg *AvrPiz-t-GFP* construct or empty vector (*GFP*) into NPB protoplasts. After 24 h incubation, the total proteins were extracted and analyzed by immunoblotting with Anti-HA or Anti-GFP antibody. The HSP protein (HSP 82) was used as a loading control.
(TIF)

S8 Fig OsAKT1-C/C1 interact with OsCIPK23. (**A**) Luciferase complementation assay for the interaction of OsAKT1-C/C1 and OsCIPK23. The indicated constructs were transiently co-expressed in *N*. *benthamiana* leaves with AvrPiz-t as negative control. Leaf disks from the leaves were used for luminescence detection 36 h after infiltration. Data are shown as means ± s.e.m. (*n* = 3). Student’s *t*-test (***P < 0.001). (**B**) Co-IP assay for OsAKT1-C/C1 and OsCIPK23 interaction. *CLuc-OsCIPK23* was co-expressed with *GUS-HA*, *OsAKT1-C* or *OsAKT1-C1* in *N*. *benthamiana* leaves. Total proteins were extracted 48 h after infiltration and subjected to immunoprecipitation.
(TIF)

S9 Fig Increasing of AvrPii protein levels doesn’t obviously affect the association of OsAKT1 and OsCIPK23 *in vitro*. The MBP-OsCIPK23-cMyc proteins were incubated with GST or GST-OsAKT1-C along with increased MBP-AvrPii-HA proteins in a pull-down assay. The input or retrieved proteins were analyzed by immunoblot with Anti-cMyc, Anti-HA and Anti-GST antibodies, respectively. The MBP-AvrPiz-t-HA protein was used as a positive control. Red arrow indicates the GST-OsAKT1-C proteins.
(TIF)

S10 Fig *OsCIPK23* expression pattern during *M*. *oryzae* infection and identification of *oscipk23* mutant. (**A**) NPB plants were sprayed with the compatible *M*. *oryzae* isolate RB22 or mock, and the shoots samples were collected at indicated time points and subjected to RNA extraction. *OsCIPK23* specific primers are used to do the qRT-PCR analysis. Data are shown as means ± s.e.m. (*n* = 3). (**B**) The schematic illustration of the structure of the *OsCIPK23* gene and the T-DNA insertion site of the *oscipk23* mutant. The black boxes indicate exons and the lines represent introns. Sites of primers used for identification of the mutant are labeled with arrows. (**C**) Genomic and transcriptional identification of the *oscipk23* mutant. The *Ubq* gene was used as an internal control.
(TIF)

S11 Fig Phenotypes of *OsCIPK23* CRISPR/Cas9 edited plants against blast inoculation. (**A**) Illustration of CRISPR/Cas9 vector targeted regions of *OsCIPK23* CDS. The red color labeled nucleotides indicate the 2 target sequences by single-guide RNAs (sgRNAs) in one construct. The underlined nucleotides indicate the protospacer adjacent motifs (PAMs). (**B**) Mutation analysis based on PCR amplication and Sanger sequencing of the genomic DNA. (**C**) Blast inoculation with a compatible isolate RO1-1 and photographed at 12 dpi. (**D**) Relative fungal growth in (**C**) were quantified by qPCR [2^[CT(*OsUbq*)-CT(*MoPot2*)]^]. Data are shown as means ± s.e.m. (*n* = 3).
(TIF)

S12 Fig High K^+^ levels inhibit blast fungal growth on medium. The *M*. *oryzae* isolates RB22 (**A**) and RO1-1 (**B**) were inoculated on the complete medium (CM) with increased K^+^ which was supplied by K_2_SO_4_. The basal CM medium contains about 18 mM K^+^. The colony diameters were measused and analyzed in the right pannel. Data are shown as means ± s.d. (*n* = 4). Different letters represent significant differences.
(TIF)

S1 Table Primers used in this study. (DOCX)
