# Supplementary figures and images for: The fungal pathogen Magnaporthe oryzae suppresses innate immunity by modulating a host potassium channel
Source: PLoS Pathog. 2018 Jan 31;14(1):e1006878. doi: 10.1371/journal.ppat.1006878 (PMC5809103; doi:10.1371/journal.ppat.1006878)

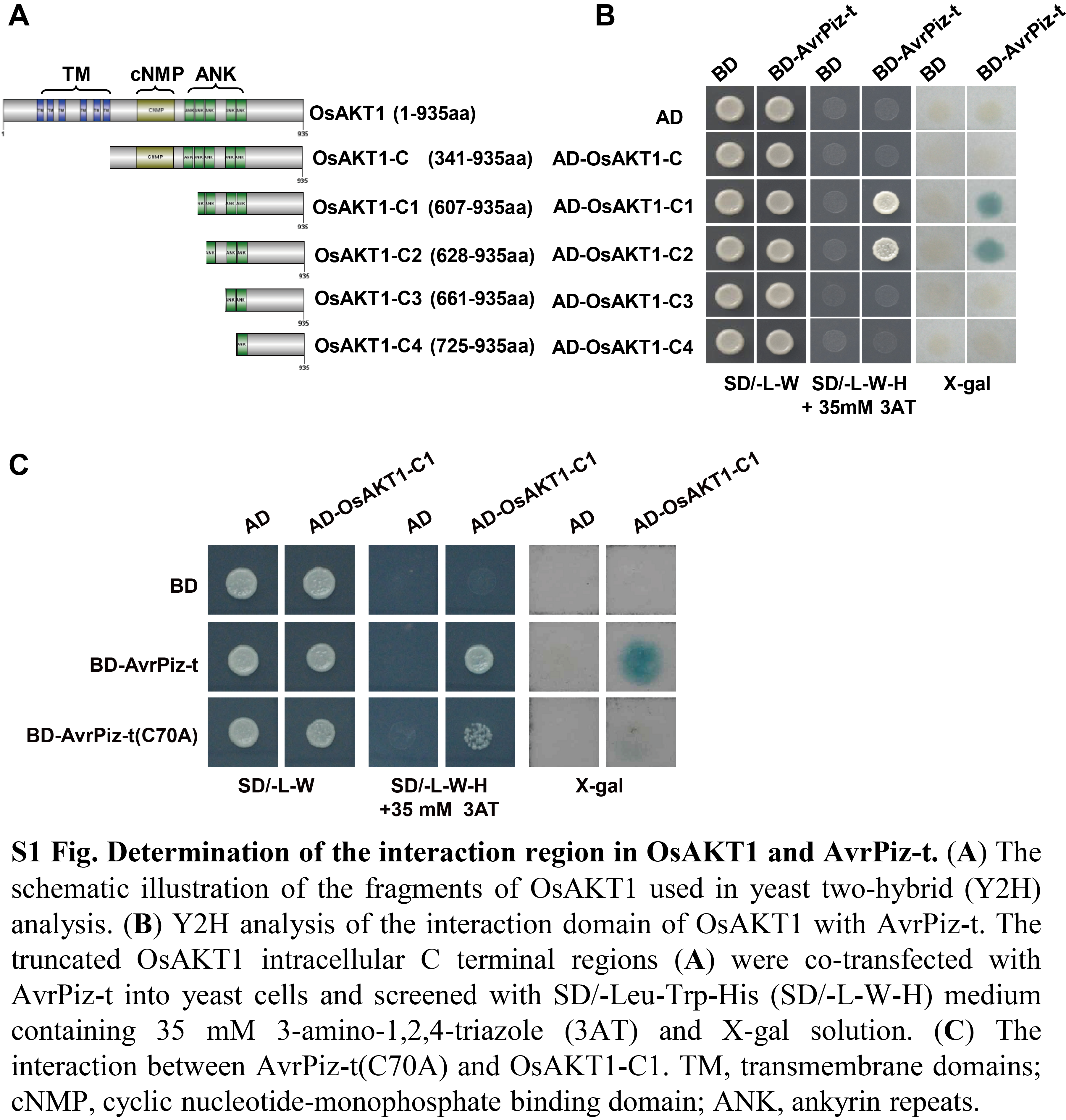

Supplement: S1 Fig — (A) The schematic illustration of the fragments of OsAKT1 used in yeast two-hybrid (Y2H) analysis. (B) Y2H analysis of the interaction domain of OsAKT1 with AvrPiz-t. The truncated OsAKT1 intracellular C terminal regions (A) were co-transfected with AvrPiz-t into yeast cells and screened with SD/-Leu-Trp-His (SD/-L-W-H) medium containing 35 mM 3-amino-1,2,4-triazole (3AT) and X-gal solution. (C) The interaction between AvrPiz-t(C70A) and OsAKT1-C1. TM, transmembrane domains; cNMP, cyclic nucleotide-monophosphate binding domain; ANK, ankyrin repeats. (TIF) [file ppat.1006878.s001.tif]

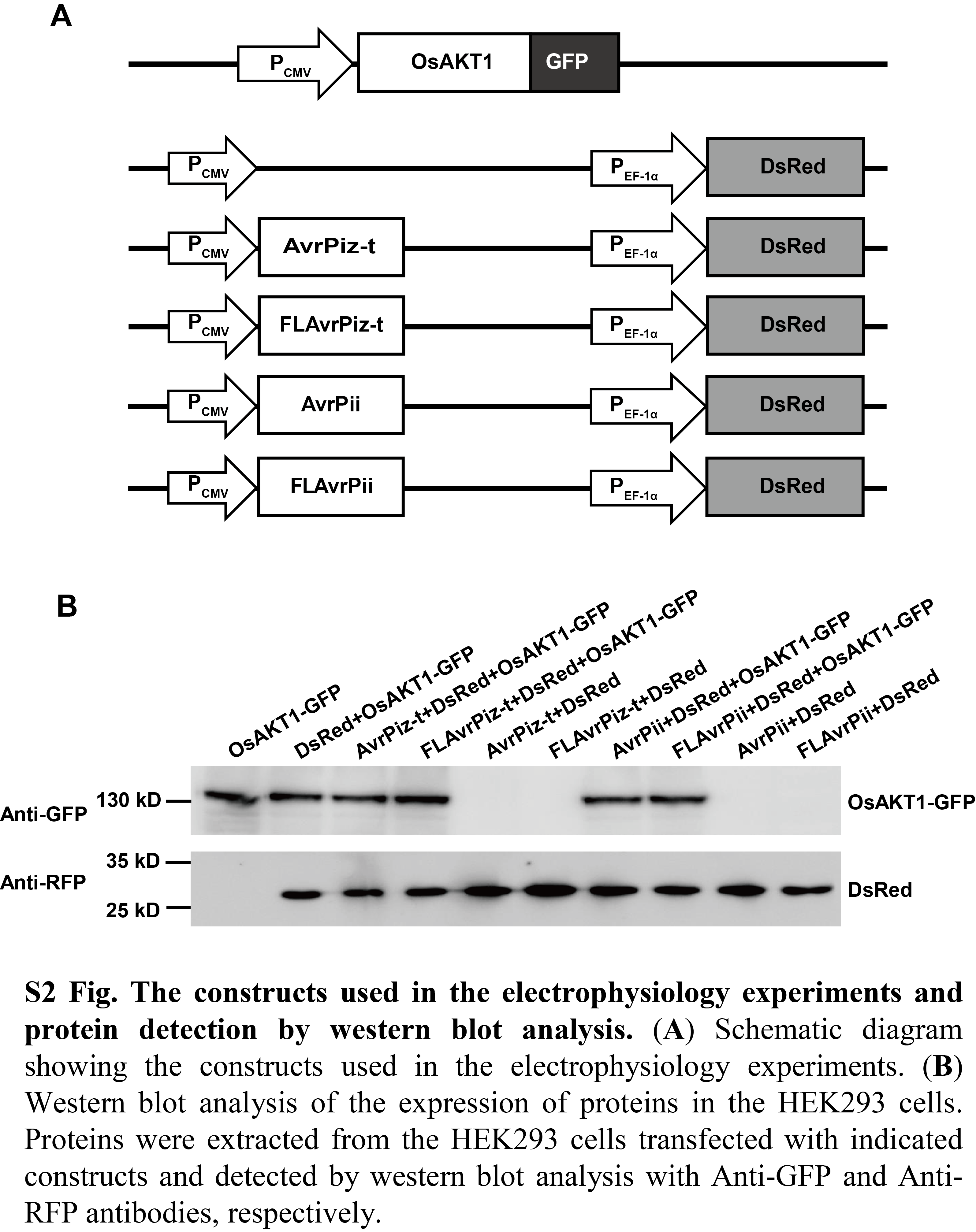

Supplement: S2 Fig — (A) Schematic diagram showing the constructs used in the electrophysiology experiments. (B) Western blot analysis of the expression of proteins in the HEK293 cells. Proteins were extracted from the HEK293 cells transfected with indicated constructs and detected by western blot analysis with Anti-GFP and Anti-RFP antibodies, respectively. (TIF) [file ppat.1006878.s002.tif]

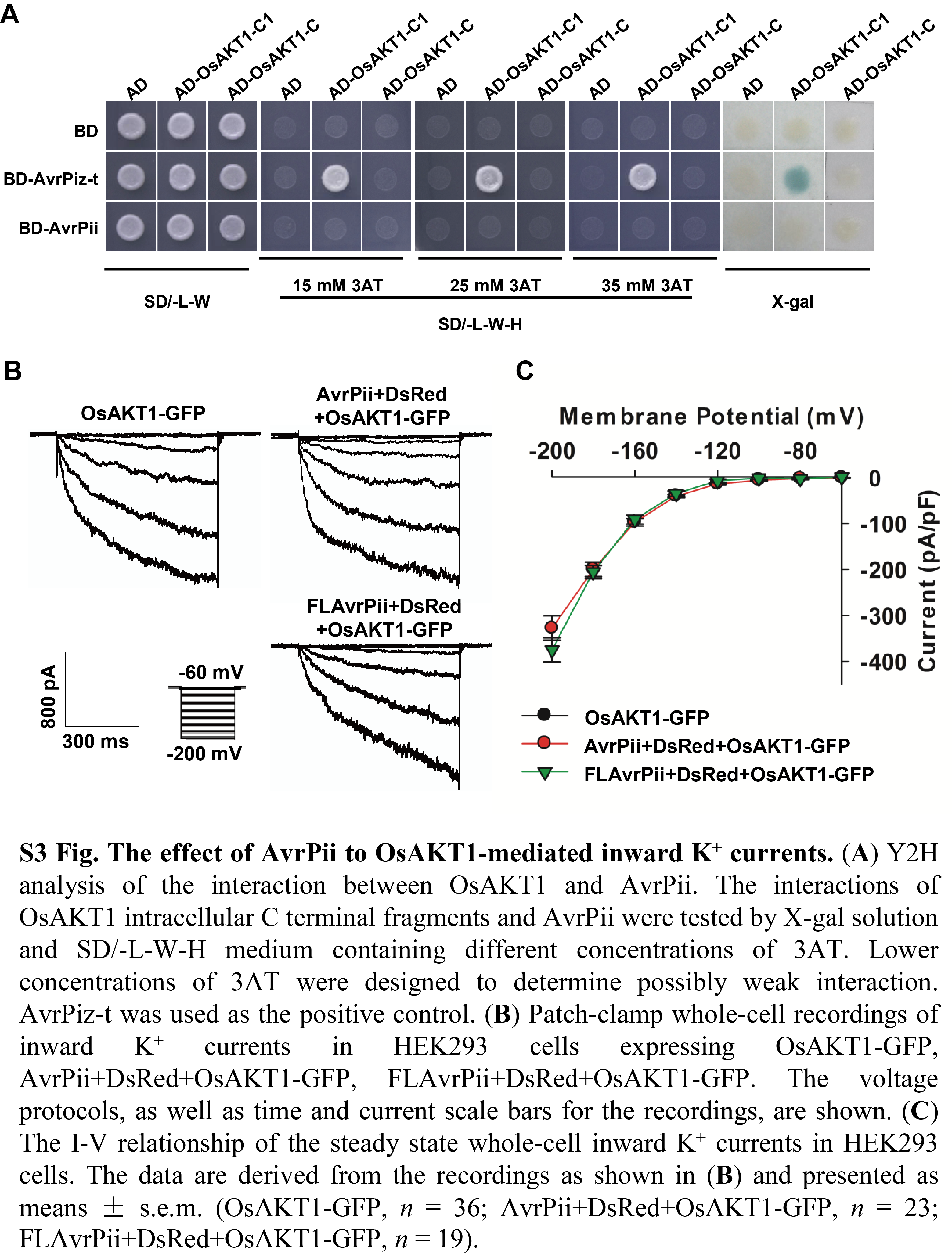

Supplement: S3 Fig — (A) Y2H analysis of the interaction between OsAKT1 and AvrPii. The interactions of OsAKT1 intracellular C terminal fragments and AvrPii were tested by X-gal solution and SD/-L-W-H medium containing different concentrations of 3AT. Lower concentrations of 3AT were designed to determine possibly weak interaction. AvrPiz-t was used as the positive control. (B) Patch-clamp whole-cell recordings of inward K+ currents in HEK293 cells expressing OsAKT1-GFP, AvrPii+DsRed+OsAKT1-GFP, FLAvrPii+DsRed+OsAKT1-GFP. The voltage protocols, as well as time and current scale bars for the recordings, are shown. (C) The I-V relationship of the steady state whole-cell inward K+ currents in HEK293 cells. The data are derived from the recordings as shown in (B) and presented as means ± s.e.m. (OsAKT1-GFP, n = 36; AvrPii+DsRed+OsAKT1-GFP, n = 23; FLAvrPii+DsRed+OsAKT1-GFP, n = 19). (TIF) [file ppat.1006878.s003.tif]

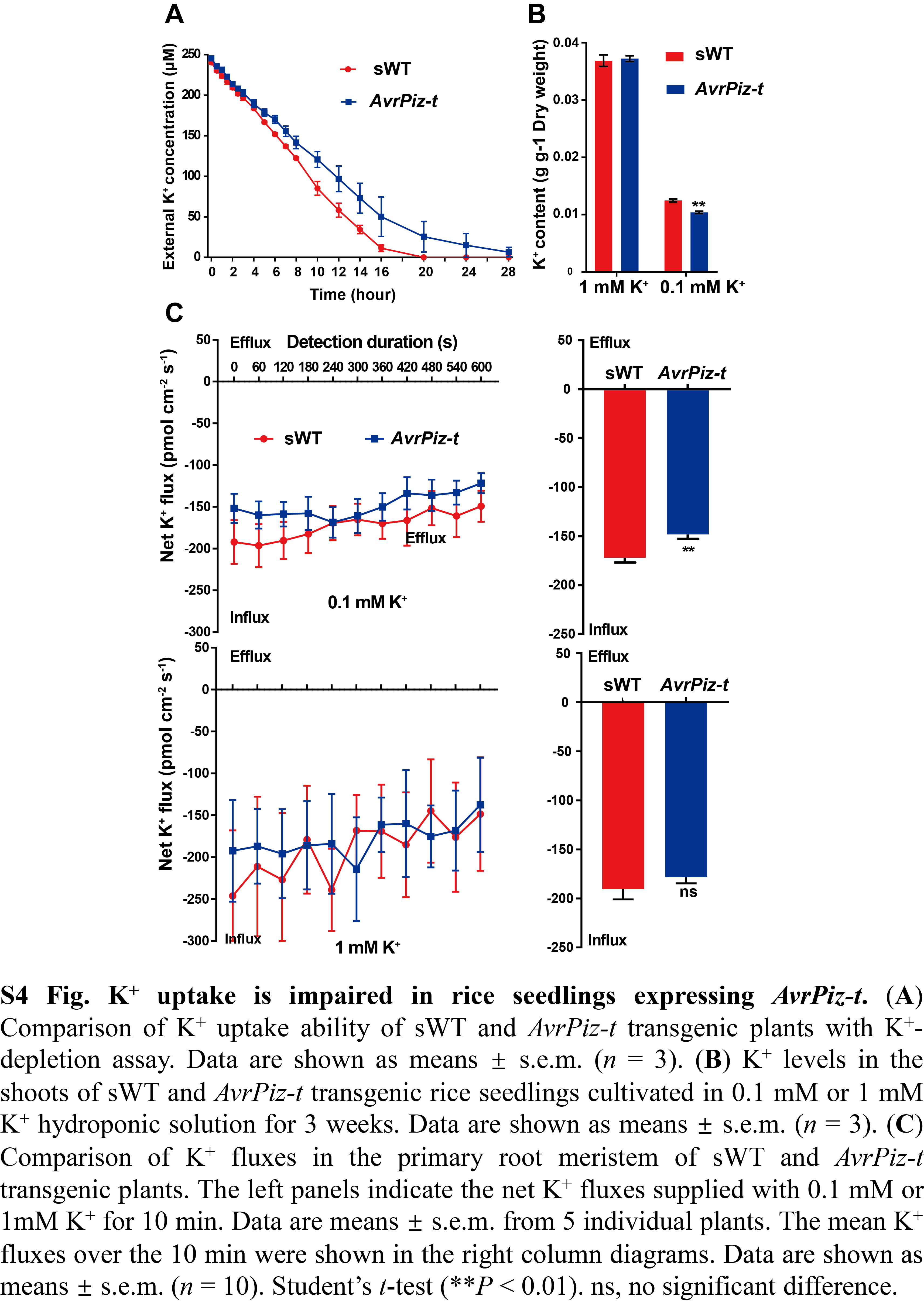

Supplement: S4 Fig — (A) Comparison of K+ uptake ability of sWT and AvrPiz-t transgenic plants with K+-depletion assay. Data are shown as means ± s.e.m. (n = 3). (B) K+ levels in the shoots of sWT and AvrPiz-t transgenic rice seedlings cultivated in 0.1 mM or 1 mM K+ hydroponic solution for 3 weeks. Data are shown as means ± s.e.m. (n = 3). (C) Comparison of K+ fluxes in the primary root meristem of sWT and AvrPiz-t transgenic plants. The left panels indicate the net K+ fluxes supplied with 0.1 mM or 1mM K+ for 10 min. Data are means ± s.e.m. from 5 individual plants. The mean K+ fluxes over the 10 min were shown in the right column diagrams. Data are shown as means ± s.e.m. (n = 10). Student’s t-test (**P < 0.01). ns, no significant difference. (TIF) [file ppat.1006878.s004.tif]

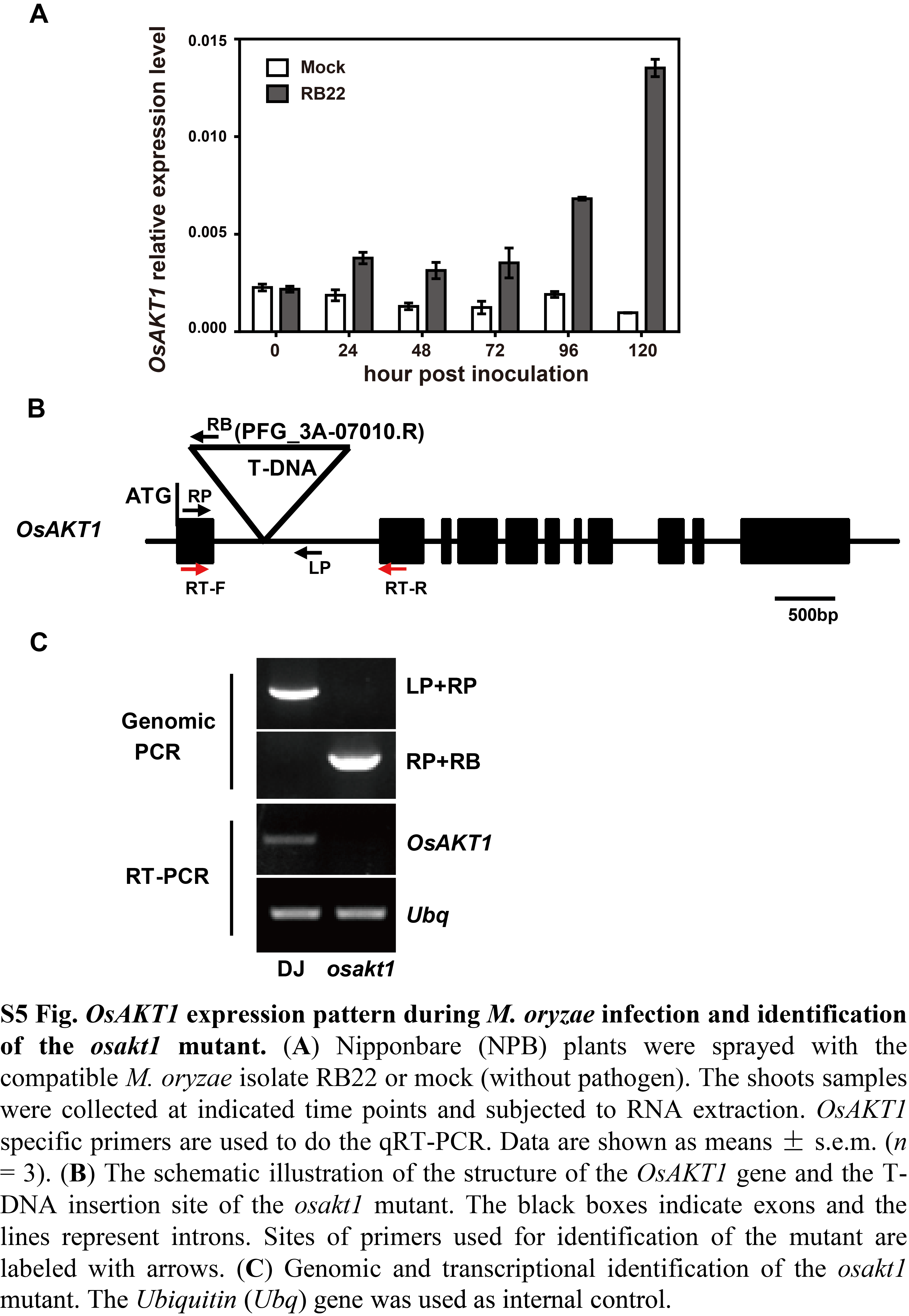

Supplement: S5 Fig — (A) Nipponbare (NPB) plants were sprayed with the compatible M. oryzae isolate RB22 or mock (without pathogen). The shoots samples were collected at indicated time points and subjected to RNA extraction. OsAKT1 specific primers are used to do the qRT-PCR. Data are shown as means ± s.e.m. (n = 3). (B) The schematic illustration of the structure of the OsAKT1 gene and the T-DNA insertion site of the osakt1 mutant. The black boxes indicate exons and the lines represent introns. Sites of primers used for identification of the mutant are labeled with arrows. (C) Genomic and transcriptional identification of the osakt1 mutant. The Ubiquitin (Ubq) gene was used as internal control. (TIF) [file ppat.1006878.s005.tif]

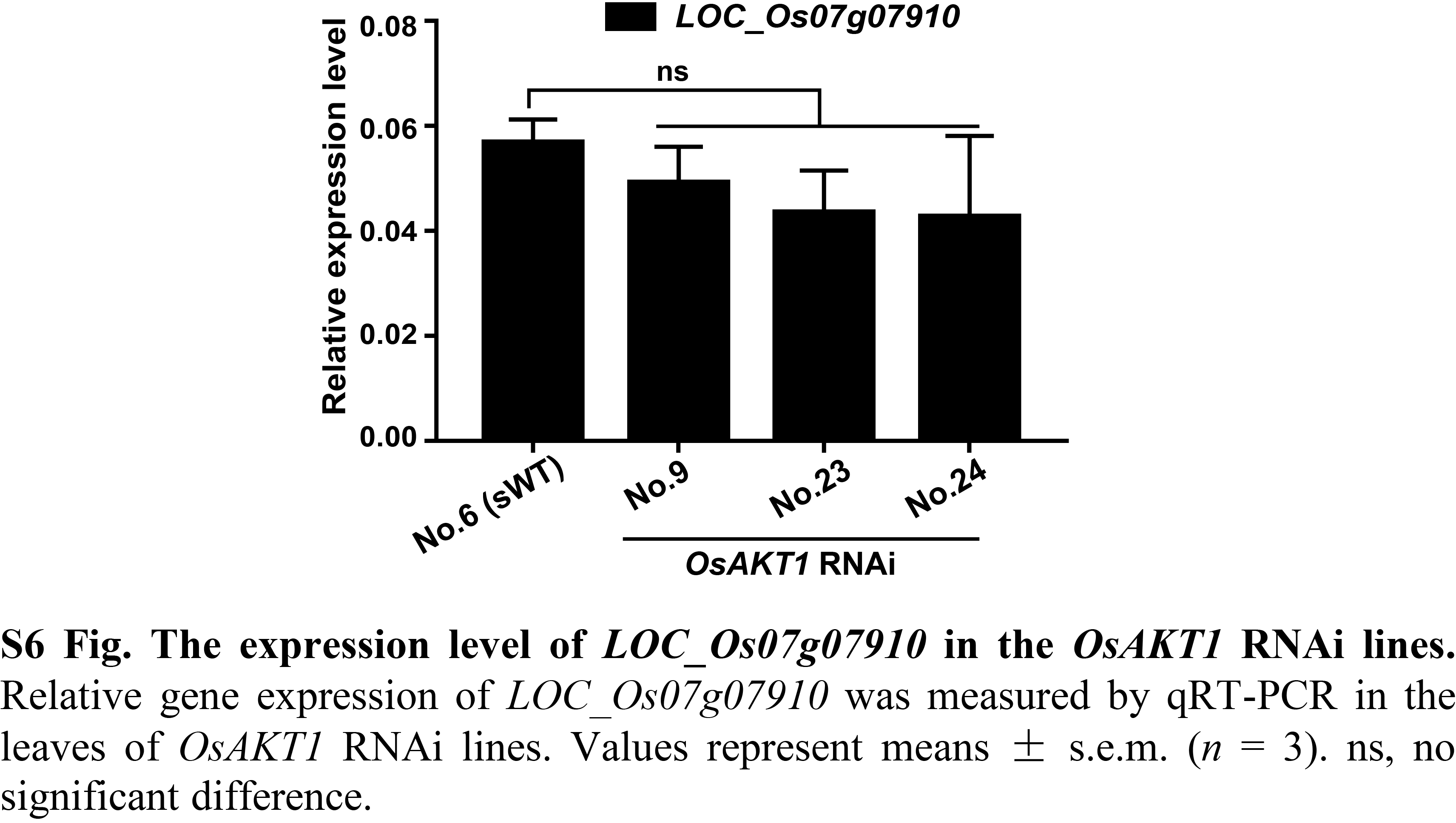

Supplement: S6 Fig — Relative gene expression of LOC_Os07g07910 was measured by qRT-PCR in the leaves of OsAKT1 RNAi lines. Values represent means ± s.e.m. (n = 3). ns, no significant difference. (TIF) [file ppat.1006878.s006.tif]

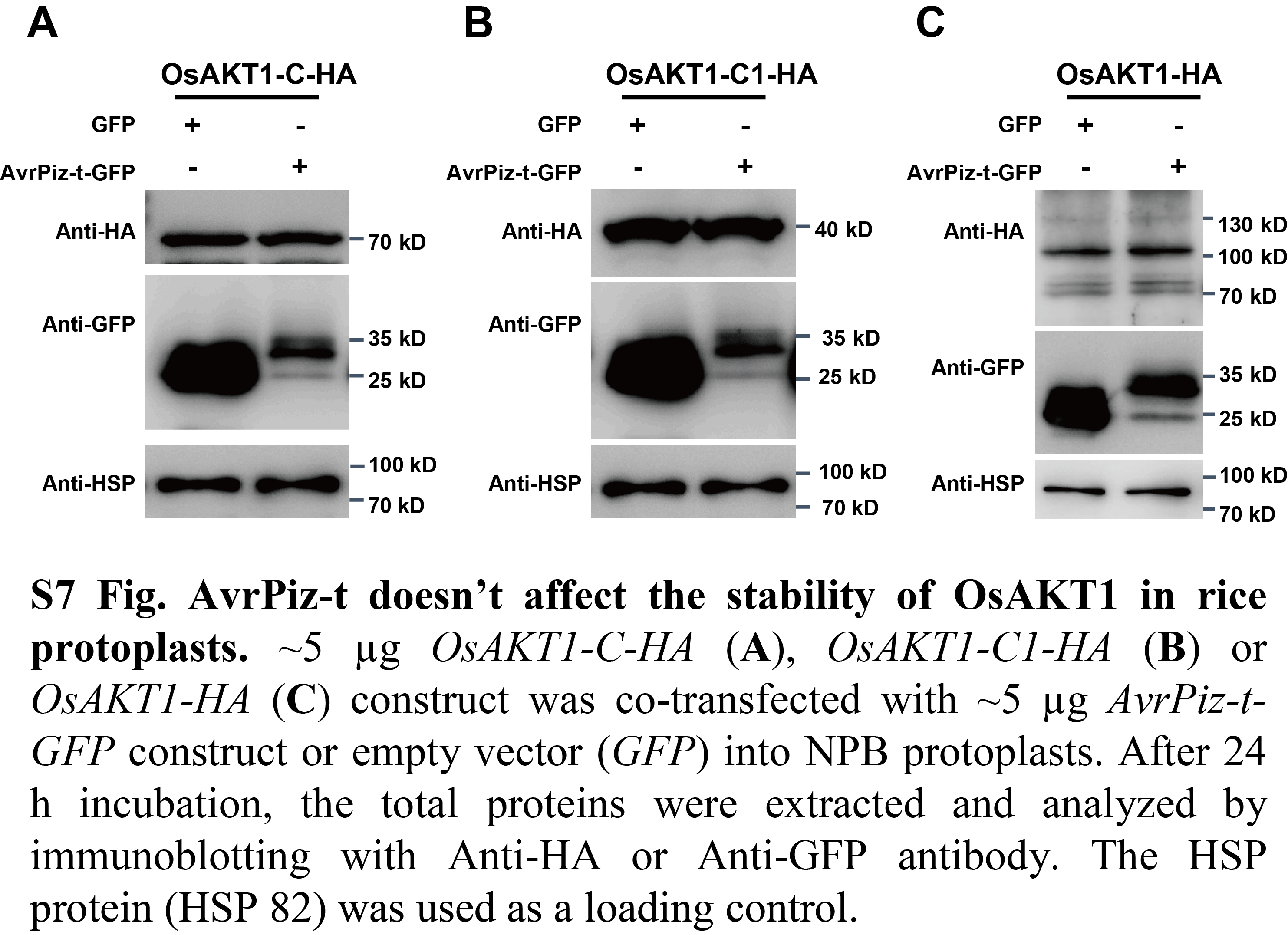

Supplement: S7 Fig — ~5 μg OsAKT1-C-HA (A), OsAKT1-C1-HA (B) or OsAKT1-HA (C) construct was co-transfected with ~5 μg AvrPiz-t-GFP construct or empty vector (GFP) into NPB protoplasts. After 24 h incubation, the total proteins were extracted and analyzed by immunoblotting with Anti-HA or Anti-GFP antibody. The HSP protein (HSP 82) was used as a loading control. (TIF) [file ppat.1006878.s007.tif]

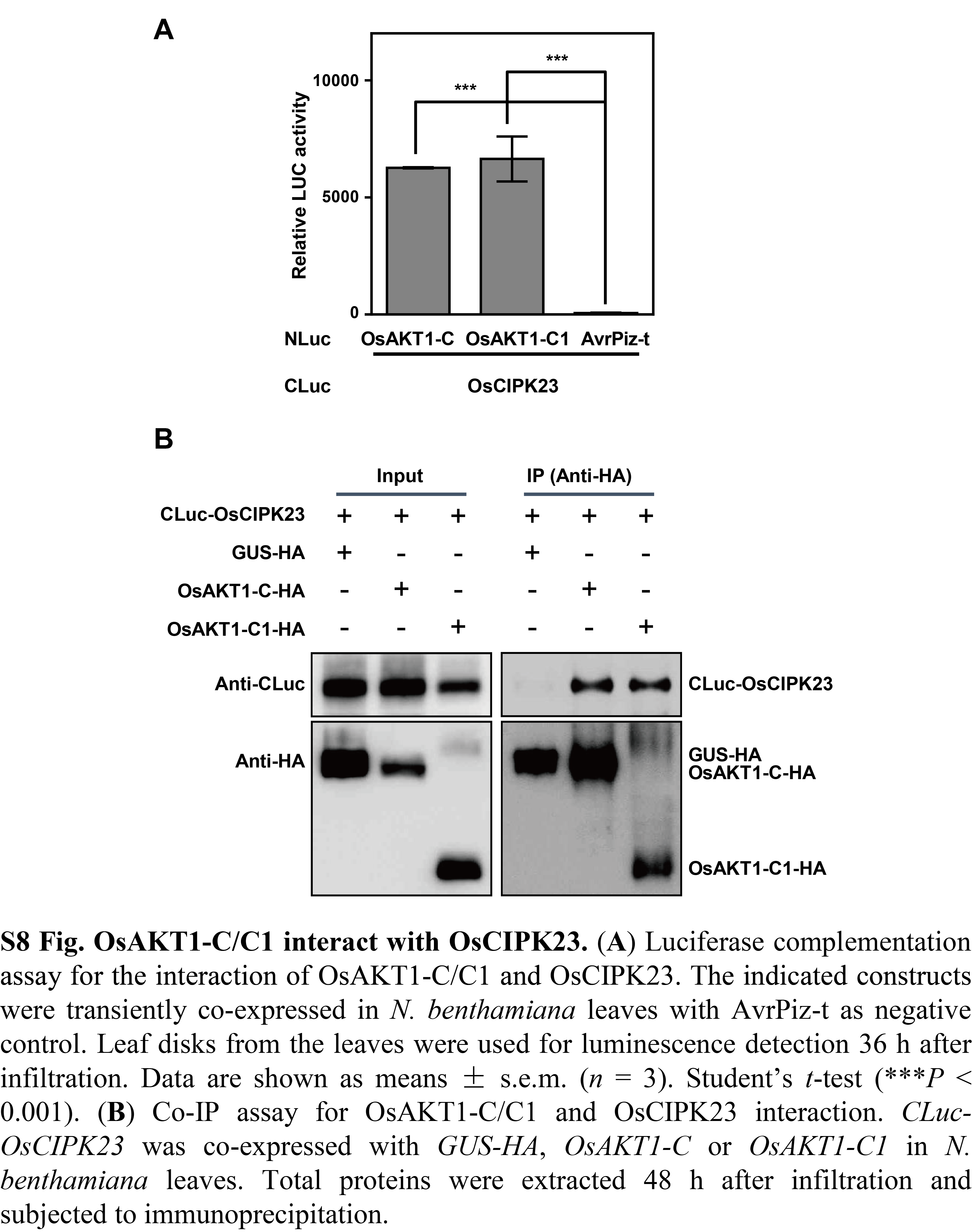

Supplement: S8 Fig — (A) Luciferase complementation assay for the interaction of OsAKT1-C/C1 and OsCIPK23. The indicated constructs were transiently co-expressed in N. benthamiana leaves with AvrPiz-t as negative control. Leaf disks from the leaves were used for luminescence detection 36 h after infiltration. Data are shown as means ± s.e.m. (n = 3). Student’s t-test (***P < 0.001). (B) Co-IP assay for OsAKT1-C/C1 and OsCIPK23 interaction. CLuc-OsCIPK23 was co-expressed with GUS-HA, OsAKT1-C or OsAKT1-C1 in N. benthamiana leaves. Total proteins were extracted 48 h after infiltration and subjected to immunoprecipitation. (TIF) [file ppat.1006878.s008.tif]

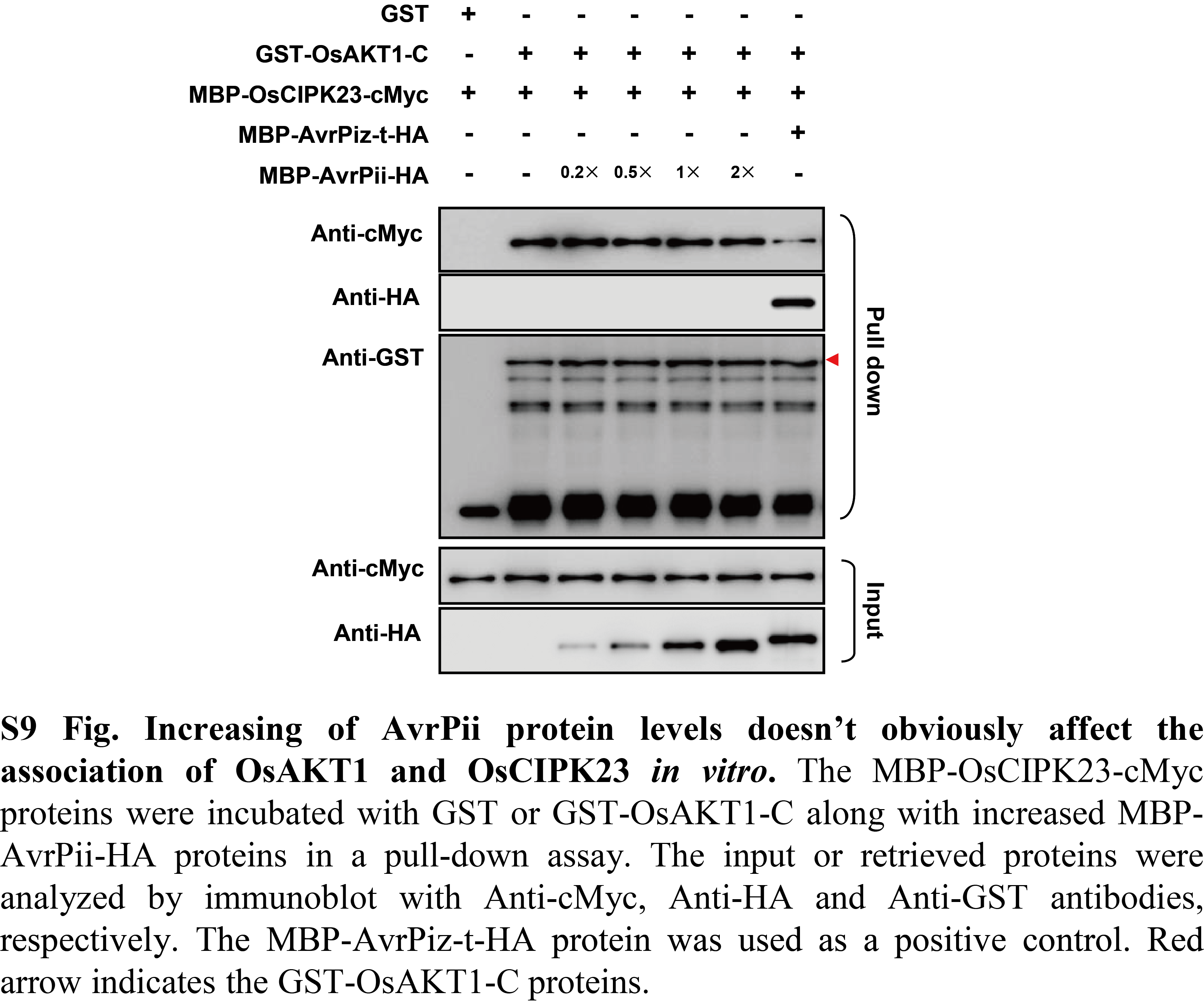

Supplement: S9 Fig — The MBP-OsCIPK23-cMyc proteins were incubated with GST or GST-OsAKT1-C along with increased MBP-AvrPii-HA proteins in a pull-down assay. The input or retrieved proteins were analyzed by immunoblot with Anti-cMyc, Anti-HA and Anti-GST antibodies, respectively. The MBP-AvrPiz-t-HA protein was used as a positive control. Red arrow indicates the GST-OsAKT1-C proteins. (TIF) [file ppat.1006878.s009.tif]

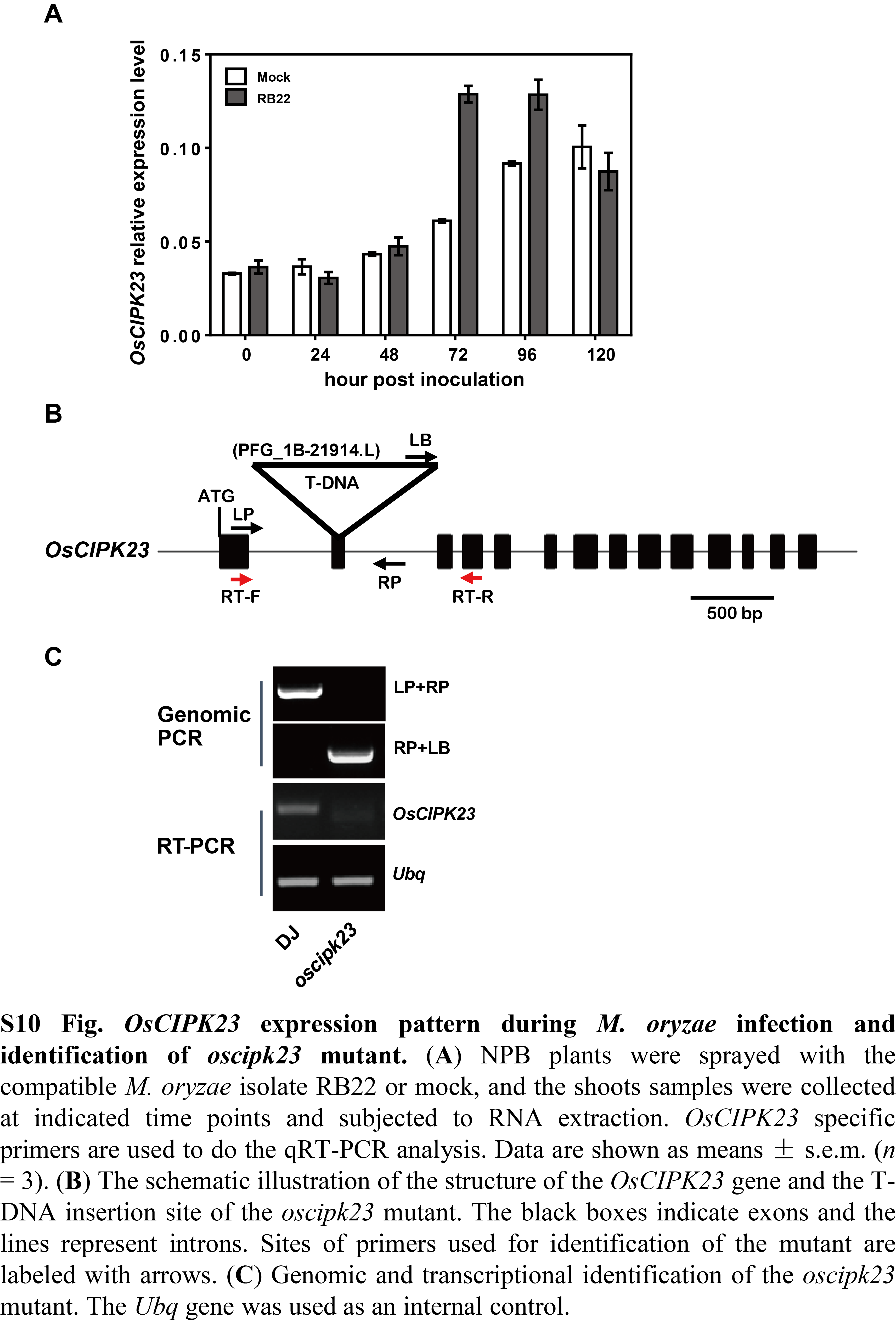

Supplement: S10 Fig — (A) NPB plants were sprayed with the compatible M. oryzae isolate RB22 or mock, and the shoots samples were collected at indicated time points and subjected to RNA extraction. OsCIPK23 specific primers are used to do the qRT-PCR analysis. Data are shown as means ± s.e.m. (n = 3). (B) The schematic illustration of the structure of the OsCIPK23 gene and the T-DNA insertion site of the oscipk23 mutant. The black boxes indicate exons and the lines represent introns. Sites of primers used for identification of the mutant are labeled with arrows. (C) Genomic and transcriptional identification of the oscipk23 mutant. The Ubq gene was used as an internal control. (TIF) [file ppat.1006878.s010.tif]

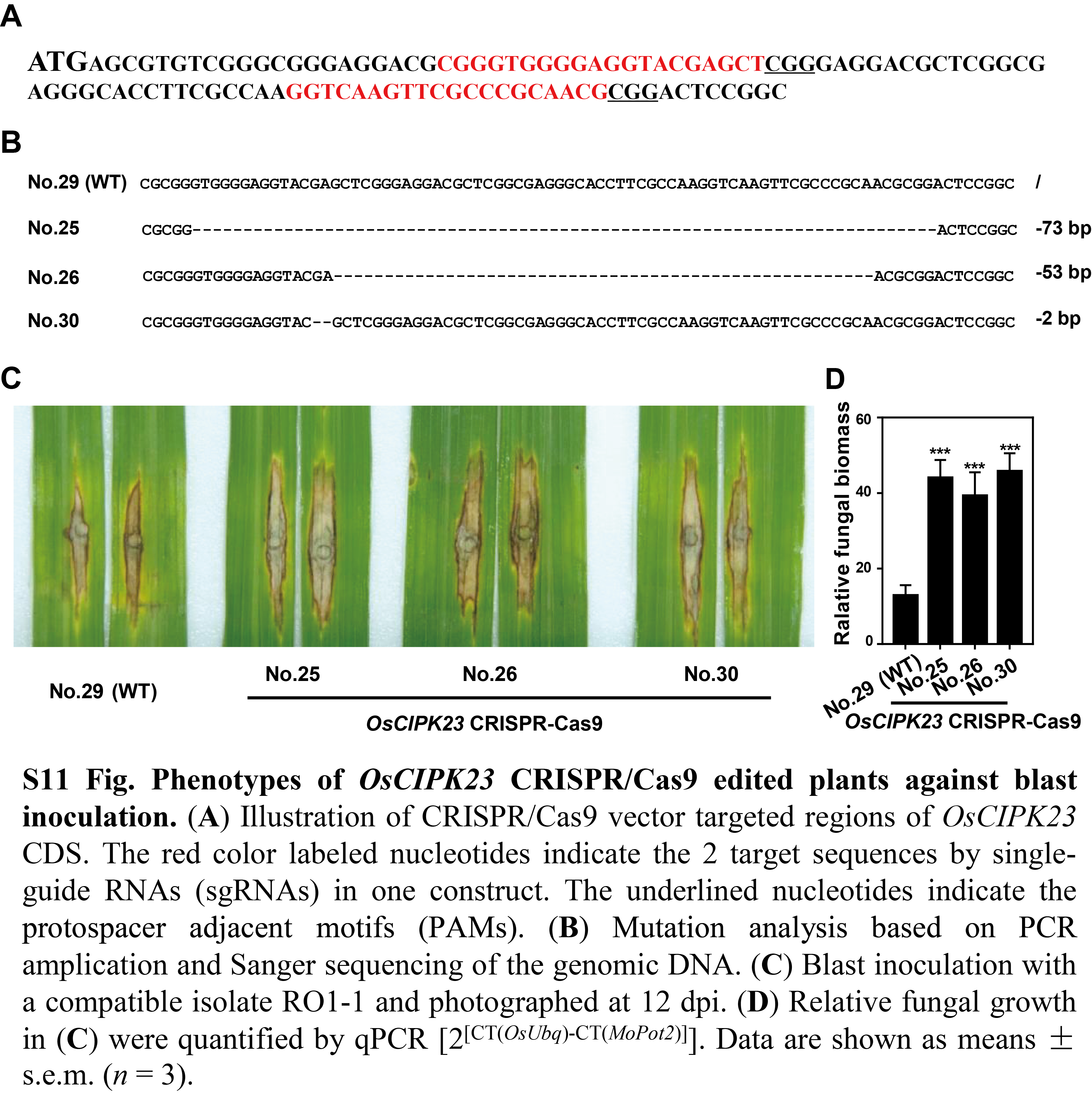

Supplement: S11 Fig — (A) Illustration of CRISPR/Cas9 vector targeted regions of OsCIPK23 CDS. The red color labeled nucleotides indicate the 2 target sequences by single-guide RNAs (sgRNAs) in one construct. The underlined nucleotides indicate the protospacer adjacent motifs (PAMs). (B) Mutation analysis based on PCR amplication and Sanger sequencing of the genomic DNA. (C) Blast inoculation with a compatible isolate RO1-1 and photographed at 12 dpi. (D) Relative fungal growth in (C) were quantified by qPCR [2[CT(OsUbq)-CT(MoPot2)]]. Data are shown as means ± s.e.m. (n = 3). (TIF) [file ppat.1006878.s011.tif]

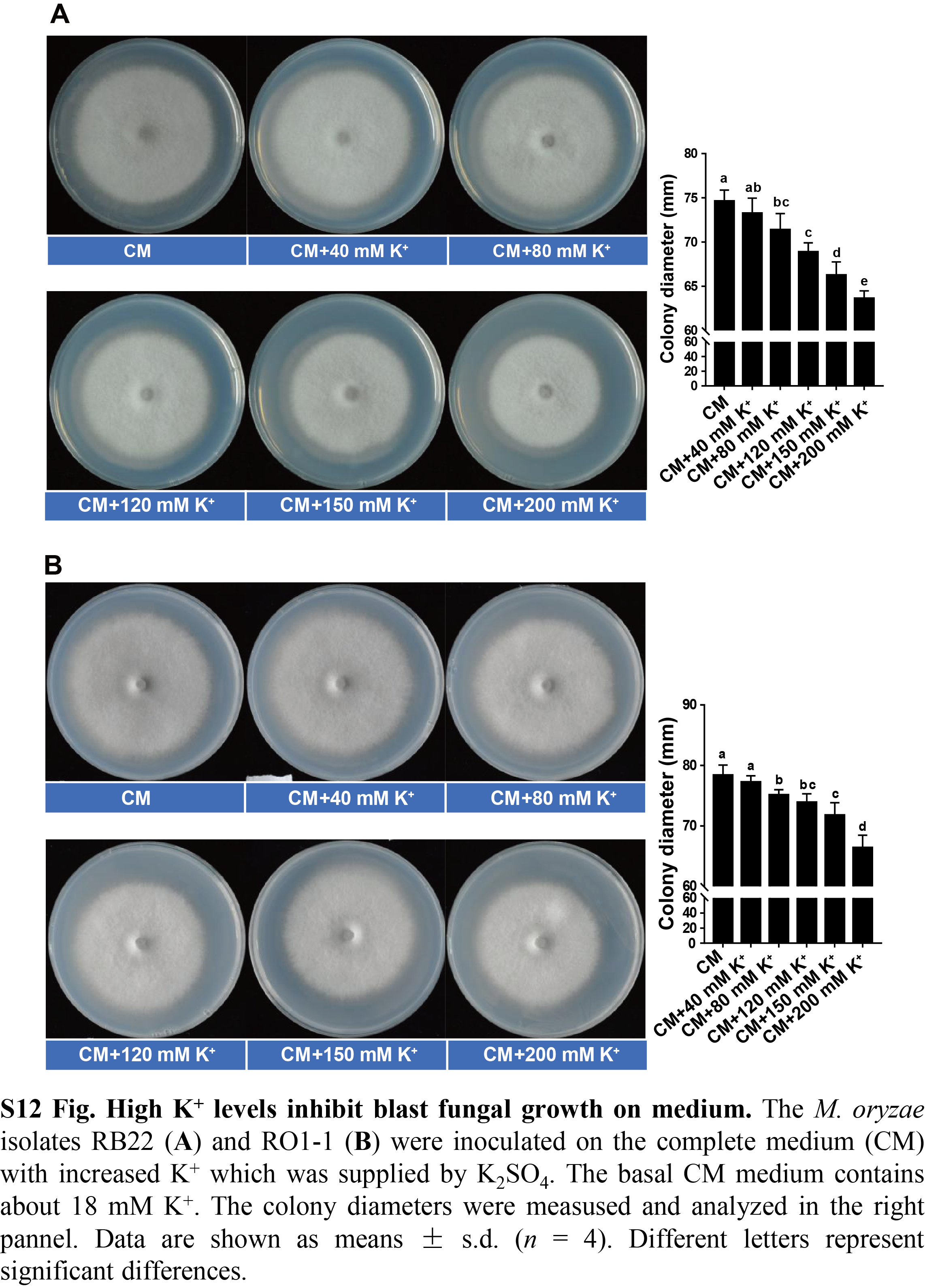

Supplement: S12 Fig — The M. oryzae isolates RB22 (A) and RO1-1 (B) were inoculated on the complete medium (CM) with increased K+ which was supplied by K2SO4. The basal CM medium contains about 18 mM K+. The colony diameters were measused and analyzed in the right pannel. Data are shown as means ± s.d. (n = 4). Different letters represent significant differences. (TIF) [file ppat.1006878.s012.tif]
