# Supplementary material for: The fungal pathogen Magnaporthe oryzae suppresses innate immunity by modulating a host potassium channel
Source: PLoS Pathog. 2018 Jan 31;14(1):e1006878. doi: 10.1371/journal.ppat.1006878 (PMC5809103; doi:10.1371/journal.ppat.1006878)
Supplement: S1 Table — (DOCX) [file ppat.1006878.s013.docx]

| **S1 Table.** **Primers used in this study**. | |
| --- | --- |
| **Primer name** | **Sequence** |
| pPC86-OsAKT1-C-SalI-F | CGGTCGACCCATGGCACTAGTCGTACT |
| pPC86-OsAKT1-C1-SalI-F | CGGTCGACCGCTTCCAAAGGAAACGAGC |
| pPC86-OsAKT1-C2-SalI-F | CGGTCGACCAGAGACTCAGAAGGAAAAG |
| pPC86-OsAKT1-C3-SalI-F | CGGTCGACCGGTGACACGGGCTTATAC |
| pPC86-OsAKT1-C4-SalI-F | CGGTCGACCCAGGACGGCAACGGCTGG |
| pPC86-OsAKT1-NotI-R | CGGCGGCCGCCTAGCTCTTGCCTTTCATC |
| pNLuc-OsAKT1-C-KpnI-F | CGGGTACCATGCATGGCACTAGTCGTACT |
| pNLuc-OsAKT1-C-SalI-R | CGGTCGACGCTCTTGCCTTTCATCTTC |
| pNLuc-OsAKT1-C1-KpnI-F | CGGGTACCATGGCTTCCAAAGGAAACGAGC |
| pNLuc-AvrPiz-t-KpnI-F | CGGGTACCATGAGCTTCGTACAATGC |
| pNLuc-AvrPiz-t-SalI-R | CGGTCGACTTGGCGCTGAGCCTGAG |
| pCLuc-AvrPiz-t-KpnI-F | CGGGTACCAGCTTCGTACAATGCAATC |
| pCLuc-AvrPiz-t-SalI-R | CGGTCGACCTATTGGCGCTGAGCCTG |
| pCLuc-OsCIPK23-KpnI-F | CGGGTACCATGAGCGTGTCGGGCG |
| pCLuc-OsCIPK23-SalI-R | CGGTCGACCGGTGACCTCCGATGCTG |
| pGEX6P-1-OsAKT1-C-EcoRI-F | CGGAATTCCATGGCACTAGTCGTACT |
| pGEX6P-1-OsAKT1-C-SalI-R | CGGTCGACCTAGCTCTTGCCTTTCATC |
| pGEX6P-1-OsAKT1-C1-EcoRI-F | CGGAATTCGCTTCCAAAGGAAACGAGC |
| pMALc2-AvrPiz-t-BamHI-F | CGGGATCCATGAGCTTCGTACAATGC |
| pMALc2-AvrPiz-t-SalI-R | CGGTCGACTCATTGGCGCTGAGCCTG |
| pMALc2-OsCIPK23-cMyc-BamHI-F | CGGGATCCATGAGCGTGTCGGGCG |
| pMALc2-OsCIPK23-cMyc-SalI-R | CGGTCGACTCAgagatcctcctcagagatgagcttctgctcCGGTGACCTCCGATG |
| pMALc2-AvrPiz-t-HA-SalI-R | CGGTCGACTCAagcgtaatctggaacatcgtatgggtaTTGGCGCTGAGCCTG |
| pMALc2-AvrPii-HA-BamHI-F | CGGGATCCATGCCCACTCCGGCCAGC |
| pMALc2-AvrPii-HA-SalI-R | CGGTCGACTCAagcgtaatctggaacatcgtatgggtaGTTGCATTTATGATTA |
| OsAKT1-C-HA-NotI-F | CGGCCCCCGCATGCATGGCACTAGTCGTACT |
| OsAKT1-C1-HA-NotI-F | CGGCGGCCGCATGGCTTCCAAAGGAAACGAGC |
| OsAKT1-C-HA-SalI-R | CGGTCGACGCTCTTGCCTTTCATCTTC |
| GUS-HA-BamHI-F | TAGAACTAGTGGATCCATGTTACGTCCTGTAG |
| GUS-HA-SalI-R | CCCCCTCGAGGTCGACTTGTTTGCCTCCCTG |
| pGDR-AvrPiz-XhoI-F | CGCTCGAGATGAGCTTCGTACAATGCA |
| pGDR-AvrPiz-t-SaII-R | CGGTCGACTTGGCGCTGAGCCTGAG |
| AvrPizt-GFP-BamHI-F | CGGGATCCATGAGCTTCGTACAATGC |
| AvrPizt-GFP-SalI-R | CGGTCGACTTGGCGCTGAGCCTGAG |
| pEGFP-N1-OsAKT1-XhoI-F | CTCGAGATGGCGAGGTGGGGC |
| pEGFP-N1-OsAKT1-HindⅢ-R | AAGCTTGCTCTTGCCTTTCATCT |
| pBudCE4.1-FLAvrPiz-t-SalI-F | CGGTCGACAATGCAGTTCTCAACCATCAT |
| pBudCE4.1-FLAvrPiz-t-BamHI-R | CGGGATCCCTATTGGCGCTGAGCCTGAG |
| pBudCE4.1-AvrPiz-t-SalI-F | CGGTCGACAATGAGCTTCGTACAATGCAA |
| pBudCE4.1-DsRed-KpnI-F | CGGGTACCATGGCCTCCTCCGAG |
| pBudCE4.1-DsRed-XhoI-R | CGCTCGAGCTACAGGAACAGGTGGTG |
| pBudCE4.1-FLAvrPii-SalI-F | CGGTCGACAATGCAACTTTCCAAAATTACT |
| pBudCE4.1-FLAvrPii-BamHI-R | CGGGATCCTTAGTTGCATTTATGATTAAAAT |
| pBudCE4.1-AvrPii-SalI-F | CGGTCGACAATGCCCACTCCGGCCAGCC |
| osakt1 3A-07010.R-LP | ATGTACTGGCCGTAACTCGG |
| osakt1 3A-07010.R-RP | GCCACTACAGCATCTCCAGC |
| oscipk23 1B-21914.L-LP | ATGTCGCCATCAAGATCCTC |
| oscipk23 1B-21914.L-RP | GGTTCACAATCTCGGCCTAG |
| 2717-NL1-LB | ATGGCAGTGAATTAACATAGC |
| Ngus-RB | AACGCTGATCAATTCCACAG |
| OsAKT1-RT-F | CGGCAGCCACTACAGCAT |
| OsAKT1-RT-R | GGCCACGTCAAGAACAAGC |
| OsCIPK23-1B-RT-F | CAAGGTCCTCAAGCACAAG |
| OsCIPK23-1B-RT-R | GTGCCACTAGCATCAAGCA |
| q-MoPot2-F | ACGACCCGTCTTTACTTATTTGG |
| q-MoPot2-R | AAGTAGCGTTGGTTTTGTTGGAT |
| q-gUBQ-F | TTCTGGTCCTTCCACTTTCAG |
| q-gUBQ-R | ACGATTGATTTAACCAGTCCATGA |
| OsAKT1-qRT-F | ATACAGAATCTGGGAGAC |
| OsAKT1-qRT-R | CATGTGGTACTATAACGC |
| OsAKT1-RNAi-BamHI-F | CGGGATCCGATCATCTTGTGCTCGTCAG |
| OsAKT1-RNAi-XhoI-R | CGCTCGAG TCTCTTCATGGTGGAATAAC |
| OsCIPK23-qRT-F | GCCTCCAAGGTTTGAGACAG |
| OsCIPK23-qRT-R | GCCAAGATTGAGACCCTGAG |
| OsPR1a-qRT-F | TCGTATGCTATGCTACGTGTTT |
| OsPR1a-qRT-R | CACTAAGCAAATACGGCTGACA |
| WRKY45-qRT-F | CGGGTAAAACGATCGAAAGA |
| WRKY45-qRT-R | GACCCCCAGCTCATAATCAA |
| UBQ-qRT-F | CGCAAGAAGAAGTGTGGTCA |
| UBQ-qRT-R | GGGAGATAACAACGGAAGCA |
| LOC_Os07g07910-qRT2-F | ACCACCTCCTCCTCGTCAC |
| LOC_Os07g07910-qRT2-R | TCTGGGTCCTTTCTGCTAA |
